# Impact of pH and salinity fluctuations on oxidation of Fe(II) by nitrate-reducing microorganisms enriched from the reduced tidal sediment of an extreme acidic river (Río Tinto, Spain)

**DOI:** 10.1093/femsec/fiaf083

**Published:** 2025-08-29

**Authors:** Martina Bottaro, Sergey Abramov, Ricardo Amils, Daniel Straub, Sebastian Kühnel, Marie Mollenkopf, Sara Kleindienst, Martin Obst, Andreas Kappler

**Affiliations:** Geomicrobiology, Department of Geosciences, University of Tübingen, 72076, Germany; Institute for Sanitary Engineering, Water Quality and Solid Waste Management, University of Stuttgart, 70569, Germany; Centro de Biología Molecular Severo Ochoa (CSIC-UAM), Universidad Autónoma de Madrid, Madrid, 28049, Spain; Quantitative Biology Center (QBiC), University of Tübingen, 72076,Germany; M3 Research Center, Medical Faculty, University of Tübingen, 72076, Germany; Geomicrobiology, Department of Geosciences, University of Tübingen, 72076, Germany; Geomicrobiology, Department of Geosciences, University of Tübingen, 72076, Germany; Institute for Sanitary Engineering, Water Quality and Solid Waste Management, University of Stuttgart, 70569, Germany; Experimentelle Biogeochemie, BayCEER, University of Bayreuth, 95448, Germany; Geomicrobiology, Department of Geosciences, University of Tübingen, 72076, Germany; Cluster of Excellence: EXC 2124: Controlling Microbes to Fight Infection, Tübingen, 72076, Germany

**Keywords:** *Denitromonas*, microbial community of the reduced sediment layer, NRFeOx microorganisms, Río Tinto tidal sediments, *Thiobacillus*

## Abstract

Nitrate reduction coupled to Fe(II) oxidation (NRFeOx) contributes to Fe cycling in the estuarian sediments of the Río Tinto river (Huelva, Spain). However, it is not yet known (i) whether and which NRFeOx microorganisms can be enriched from the reduced sediment layer and (ii) how *in situ* pH and salinity fluctuations affect NRFeOx. Therefore, we (i) used two different approaches, such as microcosm experiments (sediment amended with either NO_3_^−^/Fe^2+^_aq_ or acetate/NO_3_^−^/Fe^2+^_aq_) and enrichment cultures (medium amended with acetate/NO_3_^−^/Fe^2+^_aq_) to enrich NRFeOx microorganisms to (ii) test their salinity and pH tolerance under simulated high tide and low tide conditions. We found that different microorganisms such as *Thiobacillus* (up to 9.7 ± 5.8% DNA-based 16S rRNA gene abundance) and *Denitromonas* (83.6% DNA-based 16S rRNA gene abundance) were contributing to NRFeOx in the microcosm experiments and enrichment culture approach, respectively. The strong buffering capacity of the native sediment and the presence of additional organic carbon as acetate can favor NRFeOx microorganisms during acidic water influx (low tide) events. The ∼100% conversion of NO_3_^−^ to NO₂^−^ under high tide conditions was observed both in the enrichment cultures and microcosm experiment when acetate was added suggesting the chemodenitrification may be the primary Fe(II) oxidation pathway under salty conditions.

## Teaser

A NRFeOx enrichment culture obtained from the reduced sediment layer in RÃ­o Tinto can be active and can adapt to fluctuating pH and salinity conditions.

## Introduction

The Río Tinto is a 92-km long river that originates from the Iberian Pyrite Belt area, considered one of the biggest metal sulfide deposits within the Earth’s crust. This river is known for the high dissolved heavy metal (HM) and Fe concentrations (up to 360 mM Fe) and extreme acidity (pH ∼2.2) (López-Archilla et al. 2001, González-Toril et al. 2003), which are the result of both underground biotic activity of chemolithotrophic microorganisms (Amils et al. 2023, Vera et al. 2013) and ongoing mining activities (Olías and Nieto 2015). However, 50 km downstream (upper estuary), the influx of salt water from the Atlantic Ocean raises the river pH to circumneutral conditions (Olías et al. 2020) creating a unique geochemical environment characterized by daily pH and salinity fluctuations. The rise of pH results in the abiotic precipitation of Fe(III) (oxyhydr)oxides in the presence of O_2_ and the formation of a thick reddish “oxidized” sediment layer enriched in Fe(III) minerals, organics, and HMs (Abramov et al. 2020). Biotic Fe(II) oxidation can also contribute to Fe(III) mineral precipitation in the tidal-affected part of the river due to the presence of microaerophilic Fe(II)-oxidizing microorganisms (Abramov et al. 2021).

In Fe(III)-rich sediments, microbial Fe cycling takes place as the establishment of anoxic conditions during the high tide events transform the reddish “oxidized” sediment layer to a black “reduced” sediment through sulfate- and Fe(III)-reducing microorganisms (Abramov et al. 2021, Sanz et al. 2011). As previously described, these two distinct sediment layers are geochemically different. The reddish “oxidized” sediment (at 15 cm depth) is characterized by acidic pH and the presence of mainly Fe(III) minerals, whereas the blackish “reduced” sediment (10 cm depth) has circumneutral porewater pH, higher Fe(II) mineral content (Bottaro et al. 2025). The presence of up to 0.5 mM NO_3_^−^ in river water (Abramov et al. 2020), was shown to favor nitrate-reducing Fe oxidizing (NRFeOx) microorganisms as *Rhodanobacter*, present in the reddish “oxidized” sediment layer possibly contributing to Fe(II) oxidation and HM coprecipitation within the estuarian sediments under anoxic conditions. Under limiting organic carbon (OC), the activity of NRFeOx microorganisms as *Rhodanobacter* has been shown to promote geochemical changes, such as porewater acidification (Bottaro et al., 2025) as expected during NRFeOx (Straub et al. 1996).

NRFeOx processes have been documented in different environments, such as aquifers (Jakus et al. 2021a, Smith et al. 2017), paddy soils (Grimm et al. 2024), wastewater sludges (Pan et al. 2023), freshwater, and saltwater lake sediments (Huang et al. 2022a, Li et al. 2023). However, to the best of our knowledge, such processes have been only observed under circumneutral pH conditions (Straub et al. 1996) while acidic pH was shown to inhibit NRFeOx. The inhibitory effect is caused by the protonation of nitrite (an intermediate in denitrification) at low pH, leading to the formation of nitrous acid, which acts as a biocide for microorganisms (Jiang et al. 2011). As the porewater pH can fluctuate between 3.5 and 7 in the estuarian sediments (Bottaro et al. 2025), a negative effect on NRFeOx is expected during low tide events. Moreover, salinity increases during high tide events can also affect NRFeOx (Huang et al. 2022b).

So far, the effect of salinity and pH fluctuations on NRFeOx has not yet been studied in the black reduced sediment layer of the Río Tinto upper estuary, and no NRFeOx microorganisms were successfully isolated from this extreme environment. NRFeOx microorganisms such as *Thiobacillus* were previously detected in the *in situ* bacterial community of the “reduced” sediment layer (Bottaro et al. 2025) indicating that such microorganisms can be active in this sediment layer. Accordingly, the aims of this work were (i) to identify the microorganisms capable of NRFeOx both in microcosm experiments and enrichment cultures set up with reduced estuarian sediment of the Río Tinto river, (ii) to quantify the rates of NRFeOx in these microcosms and enrichment cultures, and (iii) to evaluate how salinity and pH fluctuations affect denitrification and favor biotic versus biotic Fe(II) oxidation in the reduced estuarian sediment of the Río Tinto river.

On a broader scale, our findings could provide insights (i) into how different commonly used enrichment approaches can affect the final NRFeOx community composition when starting from the same native sediment, (ii) into enrichment and isolation of new NRFeOx microorganisms that are tolerant to extreme pH and salinity fluctuations and (iii) into the use of these isolates for bioremediation purposes in contaminated water bodies with different geochemistry polluted by nitrate and HMs.

## Materials and methods

### Field site and sediment sampling procedure

The bulk reduced layer sediment was collected on 30 September 2022 from the upper estuary of the Río Tinto river near the city of San Juan Del Puerto during a low tide event (at 3 p.m.) at the river tidal flat (37° 18′ 39.0″ N 6° 49′ 23.0″ W). The reduced sediment layer was selected based on the black color and sulfidic smell. Geochemistry and minerology of the sediment were characterized in Bottaro et al. (2025). Sediment of the reduced sediment layer (∼10 cm depth) was collected using a sterile spatula and stored in a sterile Schott bottle under anoxic conditions in the dark at 4°C before being used for lab experiments as described in the Materials and methods sections "Microcosm experiments setup and sampling" and "Microcosm experiments: pH and salinity fluctuation setup and sampling".

### Microcosm experiments setup and sampling

The collected bulk sediment (1 kg) was mixed 1:1 with sterile anoxic low-phosphate medium (LP) buffered with 22 mM bicarbonate and prepared anoxically in a Widdel flask. The composition of the LP is described in the supporting information (Table S1). All the salts used to prepare the LP were dissolved in Milli-Q water. The pH of the medium was adjusted to 7 using sterile anoxic 1 M HCl before mixing it with the sediment. Since NRFeOx processes occur under anoxic conditions, sediment incubations were carried anoxically. Therefore, the obtained slurry was purged with a mixture of N_2_/CO_2_ (90/10) for 2 h. CO_2_ was added to the gas mixture as the used buffer for the medium was bicarbonate (Table S1). The water content of the slurry (50%) was then quantified. Subsequently, 250 ml sterile serum bottles were filled with 40 ml of slurry (with constant stirring) and 160 ml of LP to obtain a final dry sediment content of 10%. All electron donors/acceptors and C sources were added individually to each bottle depending on the setup using an N_2_ flushed gas-tight syringe next to a Bunsen burner flame to ensure anoxic and sterile setups. In four replicates per setup, one setup was amended with 1 mM lactate as NaC_3_H_5_O_3_ (hereafter lactate-amended setup) for a total of four times after every complete lactate consumption. Another setup (hereafter acetate-/NO_3_^−^-/Fe^2+^_aq_-amended setup) was amended with 0.5 mM acetate (as C_2_H_3_NaO_2_), 2 mM NO_3_^−^ (as NaNO_3_), and 2 mM Fe^2+^_aq_ (as FeCl_2_). Acetate, NO_3_^−^, and Fe^2+^_aq_ were readded four times contemporary after full acetate and NO_3_^−^ consumption. An additional setup (hereafter NO_3_^−^-/Fe^2+^_aq_-amended setup) was only amended with 2 mM of NO_3_^−^ and 2 mM of Fe^2+^_aq_ for a total of four times after full NO_3_^−^ consumption. Another setup containing acetate, lactate, NO_3_^−^, and Fe^2+^_aq_ was additionally amended with NaN_3_ to a final concentration of 160 mM (Otte et al. 2018) to inhibit microbial activity and used as abiotic control. Each preparation step was carried out under sterile conditions (under a flame) while flushing the bottles’ headspace with N_2_/CO_2_ gas (90/10 v/v), including the preparation of the solutions used as electron donors/acceptor and C sources. All microcosms were incubated anoxically (in the glovebox) at 20°C in the dark for a maximum of 73 days. The sampling was performed in an anoxic glovebox (100% N_2_ atmosphere). At each sampling event, between 1 and 2 ml of the sediment slurry was centrifuged for 10 min at 5300 × *g* and the supernatant was collected for quantification of NO_2_^−^, NO_3_^−^, total dissolved Fe, dissolved Fe(II), acetate, and lactate as well as dissolved organic carbon (DOC). The pellet formed by centrifugation was dried anoxically in the glovebox (100% N_2_ atmosphere at 18°C) until no further weight loss was observed (after 3 days) and used for sequential Fe extraction (as described in section 2.4). After each sampling, the sampled volume was replaced with the same amount of N_2_/CO_2_ gas mixture. To determine the microbial community at the beginning of the experiment, three additional bottles were prepared as previously described (without substrate addition) and used to sample 10 ml of slurry for DNA/RNA extraction. At the end of the experiment, 10 ml of slurry were sampled for each setup in triplicates, immediately frozen and stored at −80°C until DNA/RNA extraction.

### Microcosm experiments: pH and salinity fluctuation setup and sampling

After incubating the reduced sediment layer under different conditions for 73 days (as described in section 2.2), 100 ml of the remaining sediment from each bottle were equally partitioned (while working under a flame and flushing the bottles with N_2_/CO_2_ gas) in two sterile anoxic 100 ml serum bottles (50 ml of slurry for each bottle) while stirring the slurry solution. By this, two sets of two 100 ml serum bottles each containing 50 ml of sediment were prepared for each setup (maintaining the four replicates for each setup). One set of bottles was used to evaluate the salinity and the other for the pH effect on the NRFeOx microorganisms of the enrichment cultures. In the first set of bottles, the salinity was increased using a concentrated solution of 152.8 g/l NaCl and 57.4 g/l MgSO_4_ × 7 H_2_O prepared with sterile Milli-Q water. To simulate brackish water conditions, 3 ml of salt mixture (8.7 g/l NaCl and 3.3 g/l MgSO_4_) was added to each serum bottle. In the second set of bottles, sterile and anoxic 0.5 M HCl was used to lower the pH of each bottle to 6. After these first pH and salinity adjustments, all substrates were readded depending on the different setups as described in section 2.3. After full consumption of the substrates, the pH and salinity were readjusted to simulate low tide and high tide (salt water) conditions, respectively. To obtain the low tide, the pH was lowered to ∼5 using 0.5 M HCl while for high tide simulation, 3 additional ml of the concentrated salt solution were added for each bottle to obtain a final concentration of 17.4 g/l NaCl and 6.6 g/l MgSO_4_ (which is comparable to saltwater conditions). After this second pH and salinity adjustment, all respective substrates were readded in each setup. All bottles were sampled at the same time as described in the Materials and methods section "Microcosms experiment setup and sampling".

### Mineralogical analysis of sediment samples and geochemical analysis of liquid samples

For sequential Fe extractions, 2 ml of anoxic 0.5 M HCl were added to ∼200 mg of dried sample to target the poorly crystalline Fe mineral phases (Moeslundi et al. 1994). The samples were shaken for 1 h and centrifuged at 5300 × *g* for 10 min. The supernatant was collected, and 1 ml was stabilized with 1 M HCl (1:1) for Fe speciation analysis. Then, 2 ml of anoxic 6 M HCl were added to the pellet for the determination of more crystalline Fe phases (Roden and Zachara 1996). During this, the sediment was shaken for 24 h, and the supernatant was sampled as described above, followed by 1:6 dilution with 1 M HCl in the glovebox. This dilution step was necessary since Fe speciation analysis was carried outside of the glovebox and Fe(II) oxidation by O_2_ was previously observed in 6 M HCl oxic solutions (Porsch and Kappler 2011). Fe(II) and total Fe concentrations were determined immediately after stabilization of the anoxic samples using the ferrozine assay (Stookey 1970). Liquid samples for DOC analysis were preacidified with 2 M HCl (to remove inorganic carbon) and then combusted at 750°C (Elemental Analyzer, multi N/C 2100S, Analytik Jena GmbH, Germany). Fatty acid concentrations of the filtered samples were quantified by high performance liquid chromatography (class VP with a RID 10 A and PDA detector SPD-M10A VP detectors; Shimadzu, Japan). Additionally, dissolved NH_4_^+^, NO_3_^−^, and NO_2_^−^ were quantified by a segmented flow analyser (AutoAnalyzer3, SEAL Analytical, Germany) equipped with a dialysis membrane for Fe removal to prevent side reactions during measurement. Samples for Fe speciation analysis by ferrozine assay (Stookey 1970) were stabilized with 1 M HCl. In the setups containing NO_3_^−^, samples for the ferrozine assay were stabilized with 1 M HCL containing 40 mM sulfamic acid in order to prevent Fe(II) oxidation by nitrite (Schaedler et al. 2018). The pH was measured from microcosm suspensions using a benchtop pH meter (SG2, Mettler-Toledo GmbH, Germany) equipped with a pH electrode (InLab Easy DIN, Mettler-Toledo GmbH).

### NRFeOx enrichment culture setup and sampling

The NRFeOx enrichments were set up in the field using the reduced sediment layer collected as described in section 2.1. All work was done sterile next to a Bunsen burner flame. 50 g of wet sediment were mixed with 50 ml of sterile anoxic LP medium (pH adjusted to 7) in a 250 ml Schott bottle while flushing with filter-sterilized N_2_. The obtained slurry and headspace of the Schott bottle were then flushed with N_2_ for 20 min to establish anoxic conditions. Afterwards, 10 ml of the slurry were sampled with a N_2_ flushed syringe and inoculated into a 100 ml serum bottle filled with 40 ml of anoxic sterile media. The slurry was inoculated in four different 100 ml serum bottles with different media and supplement combinations: (i) LP medium (pH 7) with 2 mM Fe(II) (as FeCl_2_) and 1 mM NO_3_^−^ (as NaNO_3_), (ii) LP medium (pH 7) with 2 mM Fe(II), 0.5 mM acetate (as C_2_H_3_NaO_2_) and 1 mM NO_3_^−^, (iii) SW medium (pH 7) with 2 mM Fe(II) and 1 mM NO_3_^−^, and (iv) SW medium (pH 7) with 2 mM Fe(II), 0.5 mM acetate and 1 mM NO_3_^−^. LP refers to low phosphate medium while SW refers to sea water medium, the elemental composition of both media is provided in Table S1. Both media were buffered with a 22-mM bicarbonate buffer to a final pH of 7. After 5 days of preincubation, each 100 ml serum bottle was shaken, and 1 ml of the solution was sampled and injected in preprepared sterile anoxic Hungate tubes containing 9 ml the same medium and the same supplement combinations. From this first Hungate tube, a dilution series (10^−1^, 10^−2^, 10^−3^, 10^−4^, and 10^−5^) were prepared for each combination transferring 1 ml of the diluted slurry to a new Hungate tube with 9 ml of medium. After 30 days, a color change (the black sediment turned orange) was observed in one of the Hungate tubes with a 10^−3^ dilution supplemented with LP medium (pH adjusted to 7) and Fe(II), acetate, and NO_3_^−^. The other gradient tubes did not show any color change [attributable to Fe(II) oxidation] over time. Afterwards, 2.5 ml of the solution of the Hungate tube were Fe(II) oxidation was observed were transferred to a 50-ml serum bottle supplemented with 22.5 ml of LP medium (pH 7) and 2 mM Fe(II), 0.5 mM acetate, and 1 mM of NO_3_. Supplements were added 1 day prior inoculation since precipitation of phosphate-Fe(II) minerals was observed immediately after the addition of Fe(II). This procedure was carried out over 10 transfers every 2–4 weeks followed by incubation at 25°C in the dark. The geochemistry of the NRFeOx enrichment at transfer 10 was followed to determine NO_3_^−^ reduction and Fe(II) oxidation rates of the culture. Triplicate bottles were set up using LP medium with a pH of 6.5 supplemented with 2 mM Fe(II), 0.5 mM acetate, and 1 mM of NO_3_^−^ and 10% (v/v) of the NRFeOx culture of transfer 10. Abiotic controls were set up with the same supplements and medium but without the addition of the inoculum. For each time point, 1 ml of the bacterial culture was sampled and centrifuged for 2 min at 5300 × *g* and the supernatant was collected for determination of NO_2_^−^, NO_3_^−^, total Fe, Fe(II), and acetate. Sampling was carried out in the glovebox (100% N_2_ atmosphere) by replacing the sampled volume with the same amount of N_2_/CO_2_ gas mixture. The remaining pellet was acidified with HCl to determine the Fe(II)/Fe_tot_ ratios of the solid phase. Since nitrite formation was previously observed, 1 M HCl containing 40 mM sulfamic acid was used for pellet acidification and stabilization of the supernatant for ferrozine analysis. To determine the pH and salinity effect independently on the NRFeOx enrichment, the culture at the 17th transfer was inoculated in two different media (LP and SW) supplemented with 2 mM Fe(II), 0.5 mM acetate, and 1 mM of NO_3_. The pH of the SW medium was adjusted to 6.8 to simulate high tide conditions while different setups were prepared with the LP medium adjusted to different pH (7.0, 6.75, and 6.2). Despite the addition of the same amount of Fe(II) (2 mM), different Fe^2+^_aq_ starting concentrations were observed for each setup due to Fe(II) mineral precipitation, which generally occurs in the LP medium, but is pH-dependent with higher precipitation at circumneutral pH (Postma 1980, Hegler et al. 2008, Nordhoff et al. 2017, Goedhart et al. 2022). This would explain the lower Fe^2+^_aq_ concentrations observed at pH 7. The experimental setup and sampling were the same as previously described. For this experiment, 2 additional ml were sampled at the beginning (day 0) and at the end (day 25) for DNA extraction and 16S rRNA gene copy numbers quantification for each bottle. DNA extraction procedure is described in section 2.6. Quantitative polymerase chain reaction (qPCR) was performed for bacterial 16S rRNA genes in technical triplicates using SybrGreen Supermix (5 µl per qPCR reaction, Bio-Rad Laboratories GmbH, Munich, Germany) on a C1000 Touch thermal cycler (CFX96TM real-time system). Data were analysed using the Bio-Rad CFX Maestro 1.1, software, version 4.1 (Bio-Rad 2017).

### Microbial community analysis of the microcosm experiments and NRFeOx enrichment culture

Sediment sampling for DNA and RNA extraction at the beginning of the experiment (referred to as “initial” in Fig. 2) was carried out on three additional bottles that were set up simultaneously and identically to the other setups (as described in the section "Microcosm experiments setup and sampling") without amendments and sampled at the beginning of the experiment (day 0) to characterize the initial bacterial community of the sediment. Additionally, the same amount slurry (10 ml) was sampled at the end of the microcosm experiments (after 59 days of incubation) for all setups and controls. Simultaneous extraction of total DNA and RNA from the sediment collected from the microcosms experiments was performed using a phenol–chloroform extraction protocol (Lueders et al. 2004). The quantity and quality of the extracted DNA and RNA were determined using Qubit (Life Technologies, Carlsbad, CA, USA), NanoDrop (NanoDrop 1000, Thermo Scientific, Waltham, MA, USA), and gel electrophoresis. DNA was digested using the TURBO DNA-free™ Kit to obtain pure RNA samples, followed by reverse transcription to obtain complementary DNA (cDNA) using SuperScript™ III Reverse Transcriptase. For the NRFeOx enrichment culture, only DNA was extracted using a Power Soil DNA Kit following standard procedures provided by the manufacturer (Qiagen, Germany). 3 ml of the bacterial culture at different enrichment stages (transfer 10, 14, and 15) were sampled under anoxic and sterile conditions, centrifuged (20 238 × *g*, 2 min), the supernatant was discarded, and the pellet was stored at −20°C. The microbial 16S rRNA (gene) was amplified using primers 515F (GTGYCAGCMGCCGCGGTAA) (Parada et al. 2016) and 806R9 (GGAC TACNVGGGTWTCTAAT) (Apprill et al. 2015) targeting the V4 region. Library preparation steps (Nextera, Illumina) and sequencing were performed using Illumina MiSeq sequencing system (Illumina, USA) at the Institute for Medical Microbiology and Hygiene (MGM) of the University of Tübingen. In total 4 056 903 paired-end reads with length 250 bp were obtained for 38 samples (38 492–133 865 read pairs per sample, in average 106 761). Data processing, including quality control, reconstruction of sequences, and taxonomic annotation was done using nf-core/ampliseq version 2.10.0 (D. Straub et al. 2020) of the nf-core collection of workflows (Ewels et al. 2020). The pipeline was executed with nextflow v23.10.1 and singularity v3.8.7 (DI Tommaso et al. 2017, Kurtzer et al. 2017). Primers were trimmed using Cutadapt v4.6 (Martin 2011) and untrimmed sequences were discarded. Less than 25% of sequences were discarded per sample and 88.2% of sequences passed the filtering on average. Adapter and primer-free sequences were pooled with DADA2 v1.30.0 (Callahan et al. 2016) to eliminate PhiX contamination, trim reads (forward reads at 225 bp and reverse reads at 55 bp), discard reads with >2 expected errors, correct errors, merge read pairs, and remove PCR chimeras. Ultimately, 8162 amplicon sequencing variants (ASVs) were obtained across all samples. Between 40% and 61% reads per sample (average 56.3%) were retained. ASVs with length lower than 240 or above 270 bp were removed (5 of 8162). Taxonomic classification was performed by DADA2 and the database “Silva 138.1 prokaryotic SSU” (Quast et al. 2013). Of 8157 ASVs, 28 ASVs designated as mitochondria or chloroplasts were removed within QIIME2 version 2023.7.0 (Bolyen et al. 2019), reducing reads by <9% (average 2%). Finally, 8129 ASVs with between 17 295 and 68 201 reads (average 52 075) per sample were obtained.

### Confocal laser scanning microscopy settings, sample preparation, and imaging

Image stacks were acquired with a pixel spacing of 8.8 µm in sequential mode (Z step size 0.38 µm) using an upright Leica TCS SPE system equipped with four solid state lasers (405, 488, 561, and 635 nm) and an ACS APO 63x water immersion CS objective (NA: 1.15) (Leica Microsystems, Wetzlar, Germany). The pinhole was set to 1.0 Airy units. Transmission and reflection signals were recorded for all samples using the 488 nm laser in a detection wavelength range of 485–495 nm (Hao et al. 2016). The lectin from Glycine max (soybean) conjugated with the Alexa Fluor™ 647 (SBA) was used to stain the Extracellular Polymeric Substances (EPS) matrix, the used nucleic acids stain was SYTO 40 Blue (Molecular Probes, Inc.). The cinnamaldehyde–rhodamine derivative Fe^2+^ fluoroprobe was also used to stain the bacterial culture (Hao et al. 2016, Kumar et al. 2011). 100 µl of the bacterial culture were sampled with an anoxic N_2_ flushed syringe and stained with 1 µl of the Fe^2+^ fluoroprobe, 1 µl of SYTO^®^40 Blue diluted 1:10 in deionized water and 10 µl of 1 mg/ml Alexa Fluor™ 647 (SBA) and incubated for 15 min prior analysis. Glass slides were prepared as described by Hao et al. (2016). Blind deconvolution was applied to all 3D image stacks using the Auto QuantTM deconvolution algorithm implemented in the LEICA LAS AF software (Schmid et al. 2014) with 10 iterations. Fiji (Schindelin et al. 2012) was used for data handling and visualization. Scatterplots for correlation analysis were created using the ImageJ plugin ScatterJn (Zeitvogel and Obst 2016). The confocal laser scanning microscopy (CLSM) images shown in Fig. 4 and Fig. S7 were obtained over a volume of 345 µm^3^ and maximum intensity projections of the 3D stacks were obtained using the Z project function (after applying the despeckle filter). A fluorescence intensity threshold was selected for each individual image stack to separate signal from background noise.

## Results and discussion

### Evaluation of Fe(II) oxidation coupled to nitrate reduction in microcosm experiments—geochemistry

To investigate the activity of NRFeOx microorganisms favored by the establishment of anoxic conditions during high tide in the upper estuary of Río Tinto, the “black-reduced” sediment was amended with substrates and incubated as a microcosm for 73 days under anoxic, circumneutral pH, and brackish water conditions. The initial elemental composition of the liquid phase of these microcosm experiments is shown in Table S2. Different combinations of NO_3_^−^ (as electron acceptor), Fe^2+^_aq_ (as electron donor), and organic C sources (lactate or acetate) were added in each setup and respiked when fully consumed for a total of three to four times. An abiotic control (addition of NaN_3_) and a biotic control (no amendments) were also set up and compared to the other setups over time.

**Figure 1.**
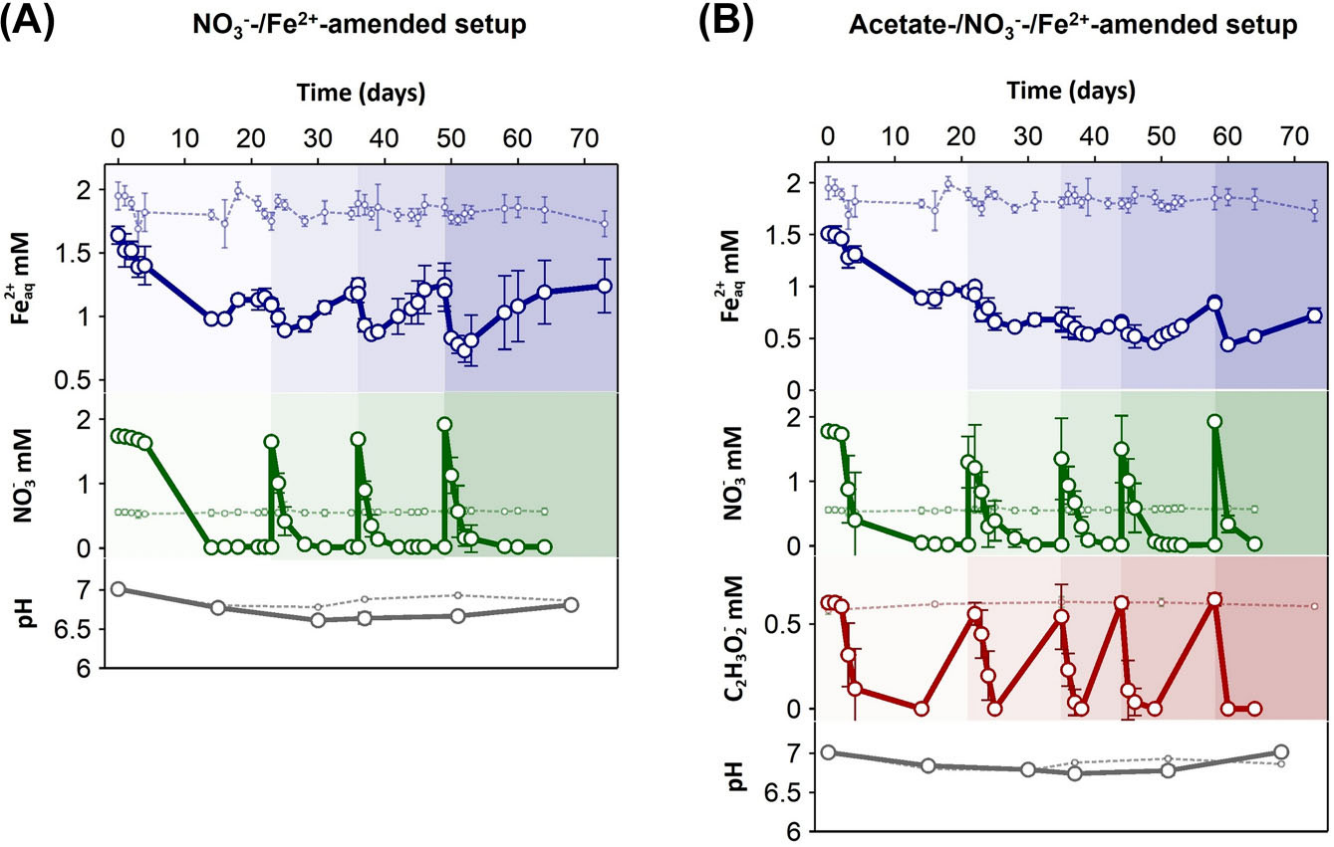
Geochemistry of microcosm experiments with black (reduced) sediments from the upper estuary of Rio Tinto. (A) Changes in NO_3_^−^ (in green), Fe^2+^_aq_ (in blue) concentrations, and pH (in gray) in the NO_3_^−^-/Fe^2+^-amended microcosms over 73 days of incubation. (B) Changes in NO_3_^−^ (in green), Fe^2+^_aq_ (in blue), acetate (in red) concentrations, and pH (in gray) over 73 days in the acetate-/NO_3_^−^-/Fe^2+^-amended microcosm. Geochemical data for the abiotic control (amended with 160 mM NaN_3_) are shown with smaller symbols in lighter color with dashed lines. Mean and standard deviation are shown for four replicates.

One setup (referred to as NO_3_^−^-/Fe^2+^_aq_-amended setup) was amended only with NO_3_^−^ and Fe^2+^_aq_ to target NRFeOx microorganisms favored by the input of Fe^2+^_aq_ (as electron donor) from the river influx or by Fe(III) reduction within the sediments. NO_3_^−^ (as electron acceptor) could be also supplied by the river or potentially produced within the sediment (as described in the introduction). To address if NRFeOx processes could take place under limiting C conditions, additional carbon sources were not added. In this setup, a simultaneous decrease of Fe^2+^_aq_ and NO_3_^−^ was observed after every NO_3_^−^ addition (Fig. 1A). The decrease of Fe^2+^_aq_ was only observed within the first 2 days after each NO_3_^−^ readdition (when NO_3_^−^ concentrations were above ∼0.3 mM, except for the first amendment) with increasing consumption rates (calculated in the first 2 days) of 0.01 ± 0.04, 0.10 ± 0.02, 0.16 ± 0.04, and 0.21 ± 0.09 mM Fe^2+^_aq_/day during the first, second, third, and fourth amendment, respectively. Within the same timeframe, nitrate consumption rates were 0.02 ± 0.04, 0.62 ± 0.11, 0.67 ± 0.09, and 0.59 ± 0.20 mM NO_3_^−^/day during the first, second, third, and fourth amendment respectively. Both Fe^2+^_aq_ and NO_3_^−^ consumption rates increased over time. After an initial decrease, Fe^2+^_aq_ concentrations kept increasing and stabilized reaching comparable values of 1.15 ± 0.07, 1.25 ± 0.05, 1.25 ± 0.17, and 1.24 ± 0.21 mM Fe^2+^_aq_ after the first, second, third, and fourth amendment, respectively, before the next NO_3_^−^ amendment. This indicates that Fe(III) reduction took place after full nitrate consumption. Overall, in the NO_3_^−^-/Fe^2+^_aq_-amended setup, the poorly crystalline Fe(III) mineral pool (extractable with 0.5 M HCl) increased over time from 0.02 ± 0.04 to 4.28 ± 3.22 mg Fe(III)/g of dry sediment within 64 days. Such an increase in poorly crystalline Fe(III) minerals was not observed in the biotic and abiotic controls. The initial pH decrease, observed for each setup and both controls, might have resulted from the mixing between the acidic sediment and the low phosphate medium. However, in the NO_3_^−^-/Fe^2+^_aq_-amended setup, the pH reached the lowest value of 6.6 ± 0.0 after 37 days of incubation, compared to the other setups and controls (Fig. S1).

The decrease of pH and the formation of poorly crystalline Fe(III) minerals, in combination with the simultaneous consumption of Fe^2+^_aq_ and NO_3_^−^, are a strong indication of on going NRFeOx (Straub et al. 1996) in the NO_3_^−^-/Fe^2+^_aq_-amended setup. NO_3_^−^ consumption could result from the combination of autotrophic NRFeOX, mixotrophic NRFeOx, or heterotrophic denitrification (Laufer et al. 2016a). Considering the low concentrations of OC in the sediment (1% C dry weight, data not shown) and the increase of OC in the dissolved phase observed in the NO_3_^−^-/Fe^2+^_aq_-amended setup (from 6.63 ± 1.01 to 9.46 ± 2.82 mg C/l in the NO_3_^−^-/Fe^2+^_aq_-amended setup; Fig. S2), we suggest that heterotrophic denitrification and mixotrophic NRFeOx did not play a major role in the NO_3_^−^-/Fe^2+^_aq_-amended setup. However, the increase in DOC concentrations could indicate DOC release and DOC consumption by denitrification and mixotrophic NRFeOx cannot be ruled out. At the same time, the Fe^2+^_aq_ oxidation extent could be underestimated due to the concomitant activity of Fe(III)-reducing microorganisms. The occurrence of Fe(III) reduction in the NO_3_^−^-/Fe^2+^_aq_-amended setup was indicated both by the increase of Fe^2+^_aq_ (observed after NO_3_^−^ consumption) and by the increase of pH at the end of the experiment (pH 7.0 ± 0.0 measured when Fe^2+^_aq_ increased up to 1.25 mM), which typically occurs during microbial Fe(III) reduction (Howell 1998). Fe(III)-reducing microorganisms can also control the availability of OC by consuming it, as it is required for their metabolism (Lentini et al. 2012) or increasing the DOC by releasing Fe(III) minerals-bound OC (Patzner et al. 2022).

The overall geochemical data of the NO_3_^−^-/Fe^2+^_aq_-amended setup suggested that NRFeOx and Fe(III) reduction were cooccurring processes. NRFeOx can first produce poorly crystalline Fe(III) minerals, which are then used as electron acceptors by Fe(III)-reducing microorganisms (Weber et al. 2006). A delayed Fe(III) reduction in the presence of NO_3_^−^ can be caused by the preferential use of nitrate as electron acceptor since it is thermodynamically more favourable (Cooper et al. 2003, Thorpe et al. 2012). Geochemical data obtained from the biotic and abiotic controls indicate a slight increase in Fe^2+^_aq_ (from 0.46 ± 0.06 mM to 0.67 ± 0.02) and no further change within 73 days of incubation, respectively. Fe extraction data targeting poorly crystalline Fe(III) minerals (extractable with 0.5 M HCl) reveals that in both controls 0.5 M HCl extractable Fe(III) was not detectable at the start of the experiment. An additional setup was amended only with lactate since lactate was detected *in situ* (not shown). In this setup, Fe^2+^_aq_ concentration initially increased (from 0.43 ± 0.03 mM to 0.61 ± 0.05 mM, within the first 14 days), similar to the biotic control, followed by a steady decrease (from 0.55 ± 0.05 mM to 0.19 ± 0.02 mM) after three consecutive additions of lactate (Fig. S3). The poorly crystalline Fe(III) phase was also close to 0 (0.9 ± 0.6 mg Fe(III)/g sediment) in this setup. Even when supplemented with lactate, known to stimulate certain Fe(III)-reducing microorganisms, the black reduced layer sediment exhibited limited Fe(III) reduction, which suggests that bioavailable Fe(III) was absent as a potential electron acceptor. We link this limitation to the absence of bioavailable Fe(III) minerals that can be used by Fe(III)-reducers as electron acceptors. Moreover, the decrease in Fe^2+^_aq_, increase of pH, production, and subsequent consumption of acetate in the lactate-amended setup suggest the occurrence of sulfate reduction (2 mM sulfate were present in the low phosphate medium) (Elliott et al. 1998, Jia et al. 2023, Luptáková et al. 2015).

One additional setup was amended with acetate, NO_3_^−^ and Fe^2+^_aq_. As described by Bottaro et al. (2025) lactate, detected in the *in situ* porewater, was transformed to acetate when the “oxidized” sediment layer was incubated anoxically. Based on these results, we concluded that acetate can be produced *in situ* and favors mixotrophic NRFeOx microorganisms. In this setup, a simultaneous decrease of Fe^2+^_aq_, NO_3_^−^, and acetate was observed after every NO_3_^−^ and acetate addition (Fig. 1B). Fe^2+^_aq_ consumption rates (calculated for the first 2 days for comparison with the NO_3_^−^-/Fe^2+^_aq_-amended setup) were 0.04 ± 0.06, 0.11 ± 0.09, 0.05 ± 0.16 and 0.06 ± 0.12 mM Fe^2+^_aq_/day during the first, second, third, and fourth amendment, respectively. Within the same timeframe, nitrate consumption rates were 0.03 ± 0.10, 0.23 ± 0.51, 0.34 ± 0.66, and 0.46 ± 0.64 mM NO_3_^−^/day. Both Fe^2+^_aq_ and NO_3_^−^ consumption rates were comparable with the ones obtained for the NO_3_^−^-/Fe^2+^_aq_-amended setups but the variation between replicates was extremely high (as it can be seen from the high standard deviations). Similarly to what we observed for the NO_3_^−^-/Fe^2+^_aq_-amended setup, after an initial decrease, Fe^2+^_aq_ steadily increased until NO_3_^−^ was readded. The increase of Fe^2+^_aq_ suggested the occurrence of microbial Fe(III) reduction as described for the NO_3_^−^-/Fe^2+^_aq_-amended setups. Overall, in the acetate-/NO_3_^−^-/Fe^2+^_aq_-amended setup, the poorly crystalline Fe(III) mineral pool (extractable with 0.5 M HCl) increased over time (from 0.00 ± 0.00 to 1.39 ± 0.99 mg Fe(III)/g of dry sediment in 64 days) yet to a smaller extent compared to the NO_3_^−^-/Fe^2+^_aq_-amended setup. The pH decreased over the course of the experiment compared to the biotic and abiotic control reaching the lowest value of 6.7 ± 0.0 after 37 days of incubation to then increased up to 7.0 ± 0.0 at the end of the experiment (pH measured after full consumption of NO_3_^−^). These values were 0.1 and 0.2 units higher than in the NO_3_^−^-/Fe^2+^_aq_-amended setup. This observation possibly indicates that Fe(III) reduction and sulfate reduction, both H+-consuming processes, might have been stimulated by the addition of acetate. NO_2_^−^ was detected after each NO_3_^−^ readdition at low concentrations with high variability between the replicates (up to 64.9 ± 40.0 µM NO_2_^−^ in the acetate-/NO_3_^−^-/Fe^2+^-amended setups) in both NO_3_^−^-/Fe^2+^- and acetate-/NO_3_^−^-/Fe^2+^-amended setups. NO_2_^−^ did not accumulate in the nitrate-amended setups (Fig. S4). The produced NO_2_^−^ can be further consumed during denitrification or by reacting with dissolved Fe(II) in an abiotic process known as chemodenitrification. However, based on the stoichiometric equation proposed by Klueglein and Kappler (2013), the amount of produced NO_2_^−^ could account for the oxidation of <0.1 mM Fe^2+^ (for each NO_3_^−^ readdition) ruling out chemodenitrification as the main Fe^2+^_aq_ oxidation pathway in the NRFeOx setups. 2. We note that NO₂^−^ concentrations may be underestimated due to its high reactivity, potentially downplaying the role of chemodenitrification.

The decrease of pH, and formation of poorly crystalline Fe(III) minerals, in combination with the simultaneous consumption of Fe^2+^_aq_ and NO_3_^−^ in the acetate-/NO_3_^−^-/Fe^2+^_aq_-amended setup (as previously observed in the NO_3_^−^-/Fe^2+^_aq_-amended setup) indicate on going NRFeOx. The lower production of poorly crystalline Fe(III) minerals and higher pH values in the acetate-/NO_3_^−^-/Fe^2+^_aq_-amended setup (compared to the setup without the addition of acetate), suggest a higher contribution of Fe(III) reduction, mixotrophic NRFeOx, or heterotrophic denitrification (as discussed for the NO_3_^−^-/Fe^2+^_aq_-amended setup) stimulated by the addition of acetate (Blöthe and Roden 2009, Calderer et al. 2010). Overall, the obtained geochemical data suggests that NRFeOx can occur within the reduced sediment layer under low and high OC conditions with comparable rates of NO_3_^−^ reduction. The activity of NRFeOx microorganisms can lead to geochemical changes, such as porewater acidification and the production of poorly crystalline Fe(III) minerals, favoring Fe cycling within the reduced sediment layer. To provide clear evidence of on going NRFeOx, we analysed the microbial composition and activity in our microcosms.

### Evaluation of Fe(II) oxidation coupled to nitrate reduction in microcosm experiments—microbial community composition, and activity

Relative abundances of microbial phyla or genera were analysed by 16S rRNA (gene) amplicon sequencing, either DNA-based (shortened to % DNA-based in the text) or RNA-based (shortened to % RNA-based in the text), to measure active key players in Fe cycling.

**Figure 2.**
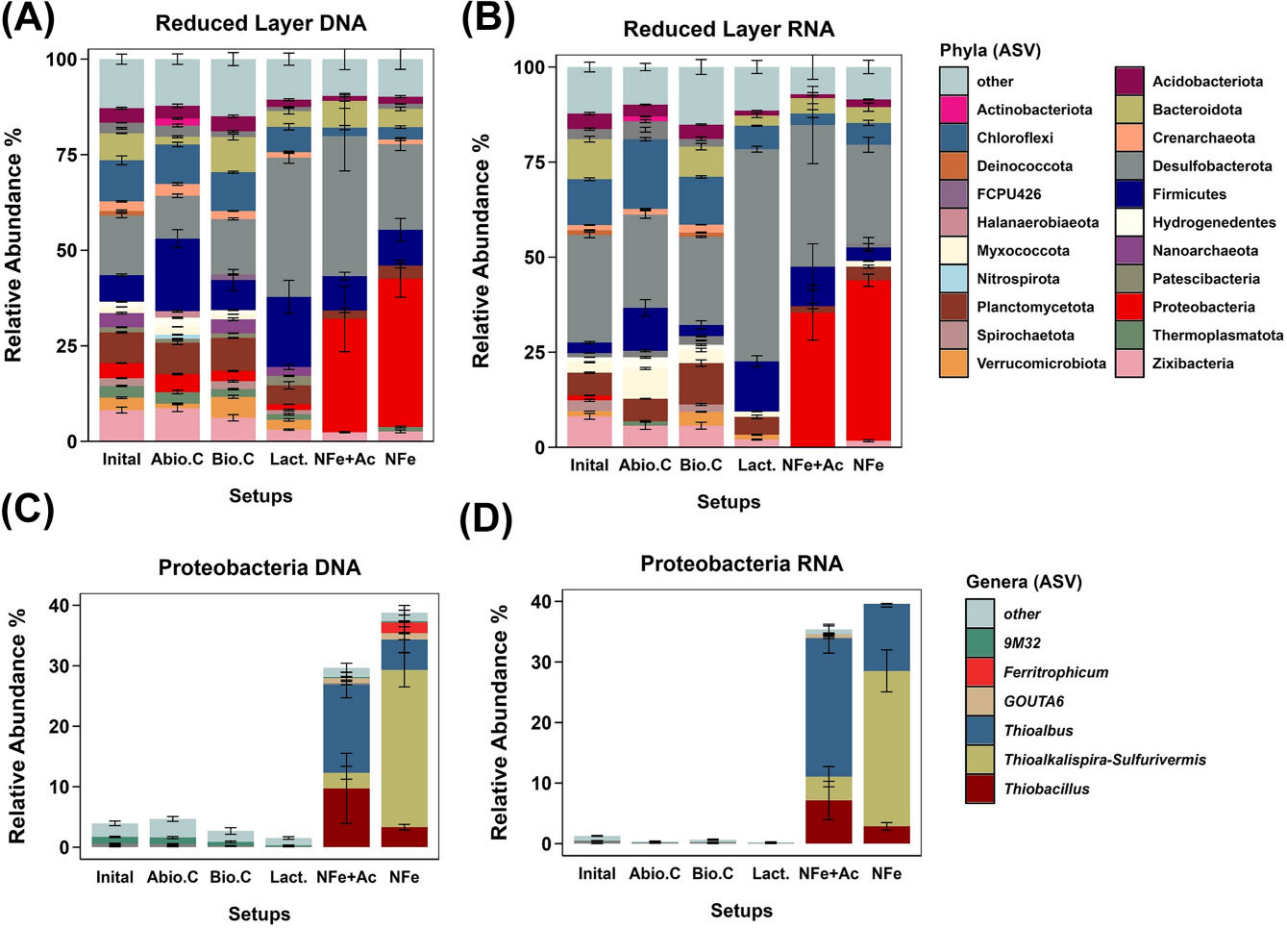
Microbial community of microcosm experiments with black (reduced) sediments from the upper estuary of Río Tinto. (A) DNA- and (B) RNA-based 16S rRNA (gene) amplicon relative abundances of selected phyla in the different setups/controls. (C) and (D) refer to DNA-based and RNA-based 16S rRNA (gene) relative abundances of the Proteobacteria phylum at a genus level. Samples for 16S rRNA (gene) amplicon sequencing were taken at different time points: day 0 (initial), and day 59 for all setups and controls. Phyla and genera with average relative abundances lower than 1% are not shown in the graphs. Bars show mean and error bars standard deviation of three replicates.

The most abundant and active phyla potentially involved in Fe cycling in the initial bacterial community in the black reduced sediment from the upper estuary of Rio Tinto (before amendment with substrates) were Desulfobacterota (15.6 ± 0.6% DNA-based and 28.2 ± 0.7% RNA-based), Bacteroidota (7.1 ± 0.1% DNA-based and 10.5 ± 0.8% RNA-based), Firmicutes (6.9 ± 0.3% DNA-based and 2.8 ± 0.2% RNA-based), and Proteobacteria (3.9 ± 0.1% DNA-based and 1.3 ± 0.1% RNA-based). Members of the Desulobacterota phyla were previously shown to drive Fe(III) mineral reduction through sulfidization (Coleman et al. 1993, Hansel et al. 2015), while members of the Bacteroidota and Firmicutes phyla can produce short-chained fatty acids during fermentation favoring Fe cycling microorganisms such as Fe(III)-reducing and sulfate-reducing microorganisms (Huang et al. 2023a, Lovley 1987, Zhang et al. 2023a). Members of the Firmicutes phylum are also capable of Fe(III) reduction (Li et al. 2011). The Proteobacteria phylum, which includes most Fe(II)-oxidizing microorganisms (Hedrich et al. 2011) as well as NRFeOx isolates and enrichment cultures (Straub et al. 2004, Kappler et al. 2005, Huang et al. 2023b), was also less abundant and active at the beginning of the experiment (Fig. 2A and B). Known NRFeOx microorganisms belonging to the phylum Proteobacteria were indeed not detectable in the initial bacterial community. Overall, the initial microbial community was dominated by Fe(III)-reducing, sulfate-reducing and fermenting microorganisms. These results matched the *in situ* microbial community data obtained from the reduced black sediment layer and trends observed in previous anoxic incubation experiments (Bottaro et al. 2025).

In the lactate-amended setups, the abundances and activities in the Desulfobacterota (36.4 ± 1.4% DNA-based and 55.7 ± 0.8% RNA-based) and the Firmicutes (18.3 ± 4.3% DNA-based and 13.2 ± 1.4% RNA-based) phyla substantially increased after 59 days of incubation. The most active genus belonging to the Desulfobacterota phylum was *Desulfobacca* (8.2 ± 0.4% RNA-based), which is a known sulfate-reducing bacterium that can utilize acetate as carbon source (Göker et al. 2011). *Clostridium in sensu stricto* (8.4 ± 2.9% DNA-based and 11.0 ± 0.9% RNA-based) was the most abundant and active genus belonging to the Firmicutes phylum enriched in the lactate-amended setup (Fig. S5C and D). Members of this genus are known fermenting microorganisms able to produce acetate (Li et al. 2023), supported by acetate appearance in the lactate-amended setup (see the section “Results and discussion”). Based on these geochemical and microbial community data, we hypothesize that in the lactate-amended setup, members of the *Clostridium in sensu stricto* genus used lactate to produce acetate favoring sulfate-reducing bacteria (SRB) such as *Desulfobacca*. Additionally, the production of iron sulfide minerals by SRB is in line with the decrease of Fe^2+^_aq_ over time (Saalfield and Bostick 2009). Therefore, in the lactate-amended setup, Fe(III) reduction was limited by the absence of poorly crystalline Fe(III) minerals (Li and Bao 2021) while fermentation and sulfate reduction were the dominant processes.

In the acetate-/NO_3_^−^-/Fe^2+^_aq_-amended setups, the increase in abundances and potential activities of the Desulfobacterota (36.6 ± 9.1% DNA-based and 37.2 ± 10.2% RNA-based) and Firmicutes phyla (9.0 ± 1.0% DNA-based and 10.3 ± 6.0% RNA-based) was comparable to what was observed in the lactate-amended setup. However, the most abundant and active genus enriched in the NO_3_^−^-/Fe^2+^_aq_-amended setup, belonging to the Desulfobacterota phylum, was *Citrifermentans* (16.5 ± 14.4% DNA-based and 11.3 ± 9.6% RNA-based) while *Bacillus* (1.4 ± 1.0% DNA-based and 8.2 ± 6.7% RNA-based) was the main enriched genus belonging to the Firmicutes phylum (Fig. S5A and B). The genera *Citrifermentans* and *Bacillus* are known as Fe(III)-reducing microorganisms (Boone et al. 1995, Yong et al. 2023, Zhang et al. 2023b). Additionally, the genus *Bacillus* has the capability of reducing nitrate in the presence of organics (Zhang et al. 2012). In the NO_3_^−^-/Fe^2+^_aq_-amended setup, the relative abundances and potential activities of the Firmicutes, Bateriodiota, and Desulfobacterota phyla did not change compared to the initial conditions. However, few genera capable of Fe(III) reduction were shown to be enriched after 59 days of incubation. Specifically, SRB bacteria such as *Desulfurivibrio* (3.1 ± 0.7% DNA-based) or *Desulfosporosinus* (1.3 ± 0.4% DNA-based) belonging to the Desulfobacterota and Firmicutes phyla, (Heidelberg et al. 2004, Sato et al. 2019) increased in the NO_3_^−^-/Fe^2+^_aq_-amended setup compared to the initial conditions and compared to the other setups. Members of the *Desulfitobacterium* genus (0.5 ± 0.2% DNA-based, belonging to the Firmicutes phylum) able to directly use Fe(III) as electron acceptor (Villemur et al. 2006), were also enriched in the NO_3_^−^-/Fe^2+^_aq_-amended setup. The identification of Fe(III)-reducing genera in both setups matched the geochemical trends (see the section “Results and discussion”).

Overall, in the microbial community of both setups with NO_3_^−^ addition (acetate-/NO_3_^−^-/Fe^2+^- and NO_3_^−^-/Fe^2+^-amended setups) the phylum Proteobacteria was the most abundant and active increasing from 3.9 ± 0.0% to 38.8 ± 4.8% in the NO_3_^−^-/Fe^2+^-amended setup and to 29.6 ± 8.6% in the acetate-/NO_3_^−^-/Fe^2+^-amended setup (DNA-based). Specifically, the main NRFeOx microorganism enriched in both setups (belonging to the Proteobacteria phylum) was the genus *Thiobacillus* (3.3 ± 0.5% DNA-based and 2.8 ± 0.6% RNA-based in the NO_3_^−^-/Fe^2+^-amended setup and 9.7 ± 5.8% DNA-based and 7.1 ± 3.1% RNA-based in the acetate-/NO_3_^−^-/Fe^2+^-amended setups (Fig. 2C and D). Members of the *Thiobacillus* genus such as *Thiobacillus denitrificans* (detected in the NO_3_^−^-/Fe^2+^-amended setup) are known (maybe autotrophic) NRFeOx microorganisms (Bosch et al. 2012) able to oxidize dissolved Fe^2+^ but also Fe(II) sulfide minerals (Fortin et al. 1996), which were previously shown to be present in the “reduced” native sediment layer (Abramov et al. 2020, Fortin et al. 1996). The activities and abundances of the genus *Thiobacillus* were, on average, higher in the acetate-/NO_3_^−^-/Fe^2+^-amended setup, as an additional 2 mM of nitrate were added compared to the NO_3_^−^-/Fe^2+^-amended setup (Fig. 1). The ability of *Thiobacillus* to reduce nitrate and oxidize Fe(II) autotrophically would explain why this genus was enriched with comparable relative abundances in NO_3_^−^-/Fe^2+^-amended microcosms both with and without addition of acetate. The genus *Ferritrophicum* (1.7 ± 2.8% DNA-based) was also shown to be enriched in the NO_3_^−^-/Fe^2+^-amended setup. This genus was shown to be physiologically very similar to other microaerophilic Fe(II)-oxidizing microorganisms (Weiss et al. 2007). However, it is not yet known if these microorganisms can also reduce nitrate.

The genus *Thioalkalispira–Sulfurivermis* (also belonging to the Proteobacteria phylum) was present in both setups amended with nitrate but mainly enriched in the NO_3_^−^-/Fe^2+^-amended setup (26 ± 3.8% DNA-based and 25.7 ± 3.4% RNA-based). This genus was previously shown to be capable of autotrophic nitrate reduction using thiosulfate or sulfide as electron donors as well as to produce and accumulate NO_2_^−^ (Sorokin et al. 2002, Wang et al. 2024a). This is supported by NO_2_^−^ presence in both NO_3_^−^-/Fe^2+^- and acetate-/NO_3_^−^-/Fe^2+^-amended setups (as discussed in the section “Results and discussion”) although NO_2_^−^ did not accumulate. NO_2_^−^ is generally produced during incomplete denitrification in OC-limited systems (Sun et al. 2009) such as in the NO_3_^−^-/Fe^2+^-amended setup or after full acetate consumption in the acetate-/NO_3_^−^-/Fe^2+^-amended setup. Small amounts of NO_2_^−^ could also be produced during NRFeOx (Jakus et al. 2021b). It has been shown that NRFeOx microorganisms such as *Thiobacillus* are also capable of using NO_2_^−^ to oxidize Fe(II) (Kelly and Wood 2000).

*Thioalbus denitrifican*s was also found abundant and active in both setups amended with nitrate. This microorganism was the main Proteobacterium enriched in the acetate-/NO_3_^−^-/Fe^2+^-amended setup (14.5 ± 2.1% DNA-based and 22.7 ± 2.4% RNA-based) and was previously described as an autotrophic nitrate-reducing, reduced-sulfur-compounds-oxidizing microorganism isolated from marine sediments (Park et al. 2011). *Thioalkalispira–Sulfurivermis* and *Thioalbus* were shown to be both capable of nitrate reduction coupled to thiosulfate or sulfide oxidation, but it is not yet known if members of these genera can also use Fe(II) as electron donor and therefore be involved in NRFeOx.

Overall, our microbial community data suggested that NRFeOx microorganisms such as *Thiobacillus* can be active in anoxic conditions in the reduced black estuarine sediments (when NO_3_^−^ is present as electron acceptor and Fe^2+^ as electron donor) even under OC-limiting conditions. Moreover, the presence of NRFeOx microorganisms can drive Fe cycling within the reduced sediment by providing poorly crystalline Fe(III) minerals and favor Fe(III)-reducing microorganisms. To assess if NRFeOx can occur under *in situ* conditions, pH and salinity were gradually adjusted in all microcosm setups to simulate high tide and low tide events.

### High tide simulation in microcosm experiments—geochemistry

High tide events in the black reduced sediment from the upper estuary of Rio Tinto were simulated by gradually increasing the salinity (in two steps, see the section “Materials and methods”) in all microcosm setups and controls to mimic brackish water and saltwater influx. All the calculated NO_3_^−^ and Fe^2+^_aq_ consumption rates, as well as NO_2_^−^ production, were compared to the results obtained during the first incubation phase (see the section “Results and discussion”).

**Figure 3.**
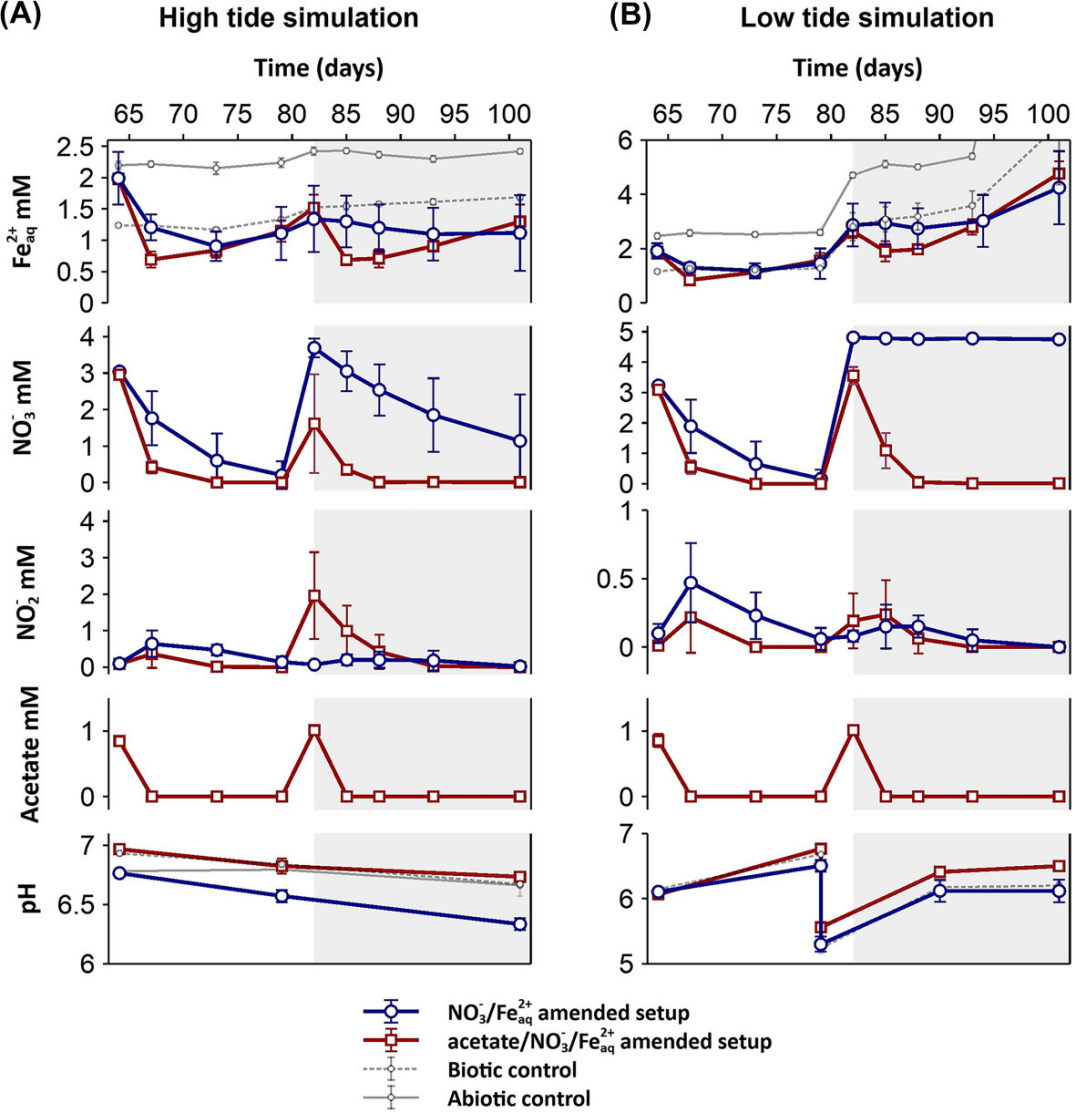
Geochemistry of microcosm experiments simulating high tide and low tide with black (reduced) sediments from the upper estuary of Rio Tinto. Changes in NO_3_^−^, NO_2_^−^, Fe^2+^_aq_, acetate, and pH in the NO_3_^−^**-**/Fe^2+^**-**amended microcosm (blue) and acetate**-**/NO_3_^−^**-**/Fe^2+^**-**amended microcosm (red) over 37 days of incubation under increasing salinity (A) and decreasing pH (B). Geochemical data for the abiotic control (amended with NaN_3_) and biotic control are included in gray dashed and solid lines, respectively. The white and gray areas in graph A indicate geochemical data collected after readdition of NO_3_^−^ under brackish (white) and salty water conditions (gray), respectively. The white and gray areas in graph B indicate geochemical data collected after readdition of NO_3_^−^ and pH adjustment to 6 (white) and 5 (gray), respectively. In both NO_3_^−^**-**/Fe^2+^**-** and acetate**-**/NO_3_^−^**-**/Fe^2+^**-**amended setups the increase of Fe^2+^_aq_ was observed after full NO_2_^−^ consumption (on day 67 for the acetate**-**/NO_3_^−^**-**/Fe^2+^**-**amended setup and on day 73 for the NO_3_^−^**-**/Fe^2+^**-**amended setup).

After the first salinity increase (day 64) simulating brackish water conditions, a simultaneous decrease of NO_3_^−^ and Fe^2+^_aq_ was observed for both NO_3_^−^-/Fe^2+^- and acetate-/NO_3_^−^-/Fe^2+^-amended setups (Fig. 3A). However, NO_3_^−^ and Fe^2+^_aq_ consumption rates were faster for the setup amended with acetate (0.85 ± 0.06 mM NO_3_^−^/day and 0.43 ± 0.05 mM Fe^2+^_aq_/day) within the first 4 days (days 64–67) compared to the NO_3_^−^-/Fe^2+^-amended setup (0.43 ± 0.25 mM NO_3_^−^ /day and 0.26 ± 0.05 mM Fe^2+^_aq_/day). For the acetate-/NO_3_^−^-/Fe^2+^-amended setup, both NO_3_^−^ and Fe^2+^_aq_ consumption rates doubled (while no changes in the NO_3_^−^-/Fe^2+^-amended setup) compared to the rates obtained during the last amendment of the incubation phase (see the section “Results and discussion”). Fe^2+^_aq_ in the biotic and abiotic control did not decrease under brackish water conditions (Fig. 3A). After this first salinity increase, 0.44 ± 0.36 and 0.36 ± 0.38 mM of NO_2_^−^ were produced in the NO_3_^−^-/Fe^2+^- and acetate-/NO_3_^−^-/Fe^2+^-amended setups, respectively. This result in a conversion of ∼13% of the initially added NO_3_^−^ to NO_2_^−^ when simulating brackish water conditions for both setups.

After a second salinity increase, simulating saltwater conditions, NO_3_^−^ and Fe^2+^_aq_ consumption rates (0.42 ± 0.45 mM NO_3_^−^/day and 0.28 ± 0.08 mM Fe^2+^_aq_/day for the acetate-/NO_3_^−^-/Fe^2+^-amended setup and 0.21 ± 0.20 mM NO_3_^−^/day and 0.01 ± 0.22 mM Fe^2+^_aq_/day for the NO_3_^−^-/Fe^2+^-amended setup) decreased compared to the rates under brackish water conditions. Fe^2+^_aq_ concentrations did not change in the NO_3_^−^-/Fe^2+^-amended setup, while NO_3_^−^ still decreased and lower amounts of NO_2_^−^ were produced (up to 0.20 ± 0.22 mM NO_2_^−^) compared to the brackish water conditions. Conversely, in the acetate-/NO_3_^−^-/Fe^2+^-amended setup, NO_2_^−^ production increased up to 1.96 ± 1.19 mM NO_2_^−^ (corresponding to 100% of the added NO_3_^−^ directly being converted to NO_2_^−^) under saltwater conditions. As previously observed, Fe^2+^_aq_ concentrations increased (from 0.69 ± 0.08 to 1.30 ± 0.27 mM) after full NO_2_^−^ consumption. Overall, the NO_3_^−^-/Fe^2+^-amended setup had the highest pH decrease of 0.4 compared to the acetate-/NO_3_^−^-/Fe^2+^-amended setup, biotic, and abiotic control) over the 37 days of incubation.

The decrease of pH and increase in Fe^2+^_aq_ and NO_3_^−^ consumption rates in the acetate-/NO_3_^−^-/Fe^2+^-amended setup suggested that brackish and saltwater conditions favored NRFeOx process possibly by inducing a shift in the microbial community at higher salinities. Specifically, Fe(III)-reducing and other denitrifying microorganisms, which can cooccur with NRFeOx processes when organics are present (as previously discussed), could be less active under halophilic conditions. Previous studies have demonstrated that increased salinity can lower the relative abundances of Fe(III)-reducing microorganisms (Bongoua-Devisme et al. 2012). Nonetheless, an increase of Fe^2+^_aq_ after full NO_3_^−^ consumption in both acetate-/NO_3_^−^-/Fe^2+^- and NO_3_^−^-/Fe^2+^-amended setups under brackish and saltwater conditions suggests that Fe(III) reduction was still occurring. Additionally, the rise in salinity can lead to the down regulation of denitrification genes (Chen et al. 2022) and to a decrease of *nirK/nirS* ratio, which promotes NO₂^-^ accumulation (Ji et al. 2018, Wang et al. 2018). A recent study showed that abundances of NRFeOx microorganisms were also significantly lower at higher salinity and chemodenitrification was the main pathway for Fe(II) oxidation under these conditions (Huang et al. 2022a). The increased production of NO_2_^−^ observed in the acetate-/NO_3_^−^-/Fe^2+^-amended setups aligns with other studies (Jiang et al. 2022a) suggesting that Fe^2+^_aq_ could be abiotically oxidized by NO_2_^−^ at higher salinities. Moreover, the decrease in denitrification extent at higher salinities, as reported in previous studies, could be supported by the decrease in NO_3_^−^ consumption rates observed in the NO_3_^−^-/Fe^2+^-amended setup under saltwater conditions, compared to what was observed in the microcosms at low salts conditions (see the section “Results and discussion”). Remarkably, in contrast to what is reported in the literature, the increase in salinity resulted in a concomitant increase of NO_3_^−^ and Fe^2+^_aq_ consumption rates, especially in the acetate-/NO_3_^−^-/Fe^2+^-amended setup. Faster denitrification rates in the acetate-/NO_3_^−^-/Fe^2+^-amended setup could be attributed to the presence of acetate (Peng et al. 2007) as well as to the higher relative abundances of NRFeOx microorganisms as *Thiobacillus* (as described in section 3.2). Overall, our data suggest that NRFeOx processes still occurred under brackish water conditions in both NO_3_^−^-/Fe^2+^- and acetate-/NO_3_^−^-/Fe^2+^-amended setups. However, under salt water conditions, NRFeOx only occurred when acetate was present (acetate-/NO_3_^−^-/Fe^2+^-amended setup), while nitrate reduction was likely decoupled from Fe(II) oxidation (since no Fe^2+^_aq_ decrease was observed) in the NO_3_^−^-/Fe^2+^-amended setup. Finally, the increased production of NO_2_^−^ suggested a higher contribution of chemodenitrification to Fe(II) oxidation under increasing salinity conditions.

### Low tide simulation in microcosm experiments—geochemistry

Low tide events in the black reduced sediment from the upper estuary of Rio Tinto were simulated by gradually decreasing the pH in two steps (see the section “Materials and methods”) to simulate the influx of acidic river water when the tides are retrieving. The calculated NO_3_^−^ and Fe^2+^_aq_ consumption rates, as well as NO_2_^−^ production, were compared to the results obtained during the pH-neutral, low-salt microcosms.

The pH was first lowered from 7 to 6 in all sediment microcosms including the controls. After this first pH decrease, at day 64, NO_3_^−^ and Fe^2+^_aq_ simultaneously decreased for both NO_3_^−^-/Fe^2+^- and acetate-/NO_3_^−^-/Fe^2+^-amended setups. However, NO_3_^−^ and Fe^2+^_aq_ consumption rates within the first 4 days (from days 64 to 67) were two times faster for the microcosms amended with acetate (0.85 ± 0.08 mM NO_3_^−^/day and 0.35 ± 0.05 mM Fe^2+^_aq_/day) compared to the NO_3_^−^-/Fe^2+^-amended microcosms (0.45 ± 0.30 mM NO_3_^−^ /day and 0.20 ± 0.11 mM Fe^2+^_aq_/day). NO_3_^−^ and Fe^2+^_aq_ consumption rates of both setups were comparable during the first salinity adjustment (Fig. 3A). Both the biotic and abiotic control did not decrease in Fe^2+^_aq_ at pH 6 (Fig. 3B). NO_2_^−^ was produced in both setups (0.47 ± 0.29 and 0.22 ± 0.23 mM NO_2_ in the NO_3_^−^-/Fe^2+^- and acetate-/NO_3_^−^-/Fe^2+^-amended setups, respectively). Overall, when the pH was adjusted to 6, NO_2_^−^ production (with concomitant Fe^2+^_aq_ decrease) as well as NO_3_^−^ and Fe^2+^_aq_ consumption rates had comparable values to the ones obtained during the first salinity adjustment (Fig. 3A) in both nitrate-amended setups.

After a second pH decrease (to pH ∼5), NO_3_^−^ and Fe^2+^_aq_ consumption rates (0.42 ± 0.45 mM NO_3_^−^/day and 0.28 ± 0.08 mM Fe^2+^_aq_/day for the acetate-/NO_3_^−^-/Fe^2+^-amended setup and 0.01 ± 0.00 mM NO_3_^−^ /day and 0.00 ± 0.37 mM Fe^2+^_aq_/day for the NO_3_^−^-/Fe^2+^-amended setup) decreased compared to the rates observed at pH 6. While for the acetate-/NO_3_^−^-/Fe^2+^-amended setup, NO_3_^−^ and Fe^2+^_aq_ were still simultaneously consumed at pH ∼5, both NO_3_^−^ and Fe^2+^_aq_ consumption stopped in the NO_3_^−^-/Fe^2+^-amended setup after the second pH adjustment (pH ∼5). In the acetate-/NO_3_^−^-/Fe^2+^-amended setup, NO_2_^−^ was still produced (up to 0.24 ± 0.25 mM NO_2_^−^) at pH ∼5, comparable to the NO_2_^−^ produced at pH 6. During this second pH adjustment, the increase of Fe^2+^_aq_ was also observed after full NO_3_^−^ and NO_2_^−^ consumption in the acetate-/NO_3_^−^-/Fe^2+^-amended setup, while for the NO_3_^−^-/Fe^2+^-amended setup, the increase of Fe^2+^_aq_ was comparable to that observed for the biotic control at pH ∼5.

From these observations, we conclude that the decrease in pH caused similar effects on Fe(II) and nitrate turnover as observed under increasing salinity in both NO_3_^−^-/Fe^2+^- and acetate-/NO_3_^−^-/Fe^2+^-amended microcosms. NO_3_^−^ and Fe^2+^_aq_ consumption rates and the concentrations of produced NO_2_^−^ were in fact similar when comparing the low tide and high tide simulation. Moreover, in the setup without amendment of acetate, NRFeOx stopped both under saltwater and low pH conditions. As observed in the pH-adjusted microcosms (Fig. 3B), upon acidification, Fe^2+^_aq_ increased in all the setups and controls, probably due to Fe(II) mineral dissolution (Voelz et al. 2019). Therefore, the influx of acidic water after the retrieval of the tides could provide Fe^2+^_aq_ for NRFeOx. Moreover, the rapid increase of pH observed after each pH adjustment (Fig. S6) indicates a strong buffering capacity of the native sediment potentially beneficial for NRFeOx microorganisms. NRFeOx microorganisms are in fact known to thrive best in circumneutral environments (Laufer et al. 2016, Straub et al. 2004) and their activity can be negatively affected by a decrease in pH (Jiang et al. 2022b). However, to the best of our knowledge, no data are available on the activity of NRFeOx microorganisms at the pH range tested in our low tide simulation experiment (between pH of 5 and 6). Denitrification rates generally decrease at pH below 6 (Saleh-Lakha et al. 2009) but bacterial communities can adapt to low pH conditions through acid-tolerant denitrifying bacteria capable to lower their pH optimum (Parkin et al. 1985). This adaptation might explain the increased nitrate consumption rates observed in both the acetate-/NO_3_^−^-/Fe^2+^- and NO_3_^−^-/Fe^2+^-amended setups at pH 6 and in the acetate-/NO_3_^−^-/Fe^2+^-amended setup at pH ∼5. Moreover, NRFeOx microorganisms grown with acetate were linked to a higher tolerance to lower pH (Straub et al. 2004), which could explain why NRFeOx processes were only inhibited in the NO_3_^−^-/Fe^2+^-amended setup at pH 5. The production of NO_2_^−^ observed in the low tide experiment suggests that chemodenitrification was a major driver of Fe(II) oxidation at pH below 7, similarly to what we observed at higher salinities. The pH effect on chemodenitrification will be further discussed in the next sections.

Overall, using microcosm experiments, we found that NRFeOx microorganisms are present within the black reduced sediment layer from the upper estuary of Rio Tinto and NRFeOx can still occur under low and high tide conditions. Moreover, the native bacterial community seemed to be adapted to salty and more acidic conditions since the NRFeOx were shown to be faster (compared to the first enrichment phase) especially when additional OC was present. Finally, the analysis of pH over time suggests that the strong buffering capacity of the native sediment can “protect” NRFeOx microorganisms during acidic water influx events.

### Enrichment of NRFeOx from the reduced tidal sediment—microbial community composition and physiology

**Figure 4.**
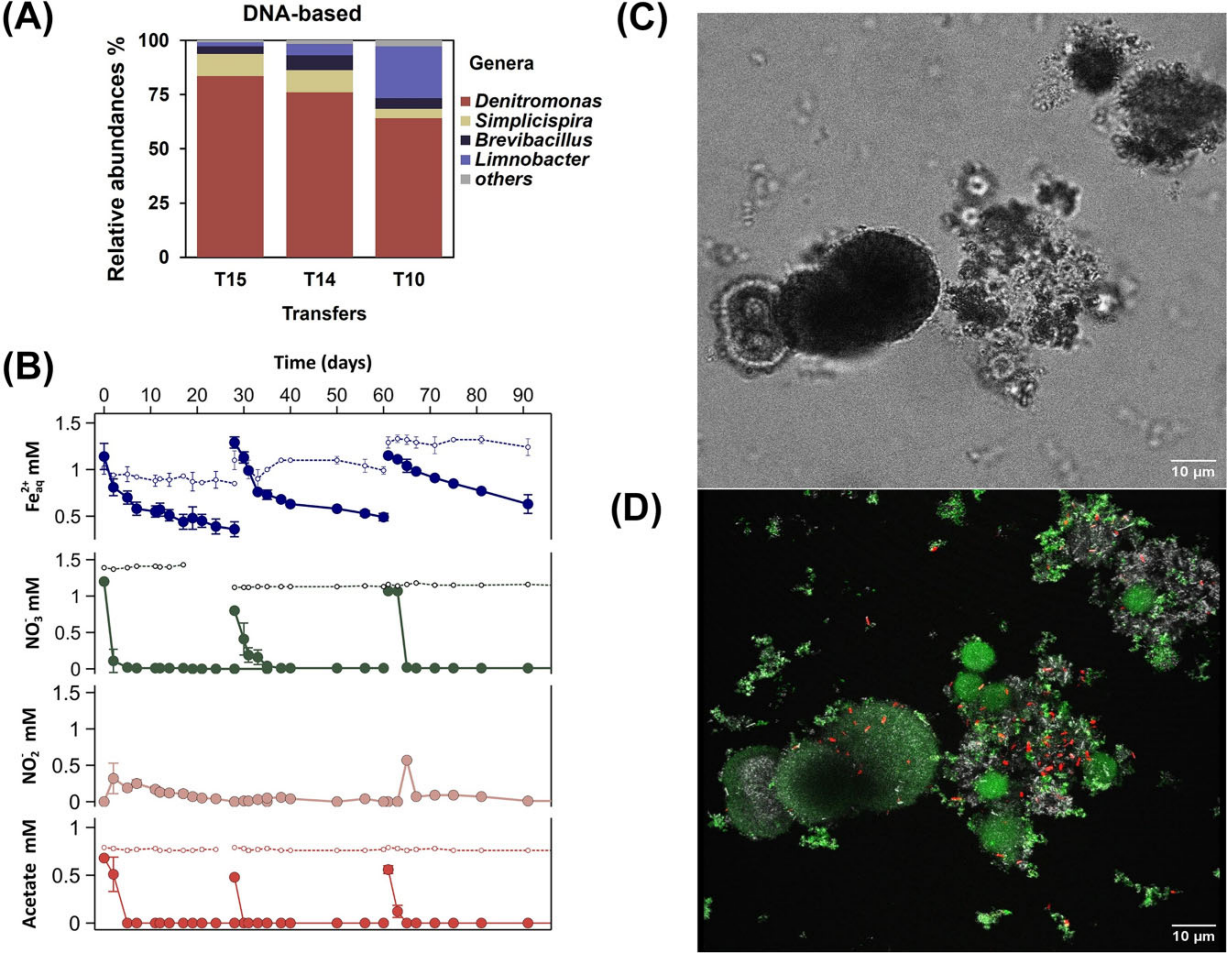
Geochemistry, microbial community, and agglomerate structure of the NRFeOx enrichment culture. (A) DNA-based 16S rRNA gene relative abundances of the main genera present in the NRFeOx enrichment culture. (B) Changes in NO_3_^−^ (green), NO_2_^−^ (orange), Fe^2+^_aq_ (blue), and acetate (red) concentrations in the NRFeOx culture grown in three consecutive transfers. (C) Transmission image of the NRFeOx enrichment and (D) maximum intensity projection of a 3D stack of the same spot to visualize the EPS (in green, SBA 647), DNA (in red, SYTO 40), and mineral components (in gray). The staining procedure, image acquisition and treatment are described in section 2.7.

NRFeOx enrichment cultures from the estuarian black reduced sediment were inoculated in the field, kept at room temperature in the dark, and transferred to fresh medium after 4 weeks when Fe(II) oxidation was visually observed (when orange precipitates were visible). Enrichment setups consisted of Fe(II) (2 mM) and nitrate (1 mM), with or without acetate (0.5 mM). None of the bottles incubated with Fe(II)/nitrate and only a few bottles incubated with Fe(II), nitrate, and acetate showed Fe(II) oxidation. One of these successful enrichments was selected for further transfers and characterization. After 10, 14, and 15 transfers (indicated as T10, T14, and T15 in Fig. 4A) the bacterial culture was sampled for DNA extraction and 16S rRNA gene amplicon sequencing.

Sequencing revealed a community dominated by Gammaproteobacteria, with the highest relative abundance of bacteria belonging to the *Denitromonas* genus (Fig. 4A). Members of this genus were described as denitrifying bacteria present in marine microbial communities (Wang et al. 2024b) and previously enriched from sediment collected in a Danish Fjord upon addition of Fe(II), nitrate and acetate (Laufer et al. 2016). However, to the best of our knowledge, there is no evidence that this genus can reduce nitrate by coupling it to Fe(II) oxidation. Other abundant genera belonging to the Gammaproteobacteria as *Limnobacter* (up to 23%) and *Simplicispira* (up to 10%) were also enriched. The genus *Brevibacillus* (up to 6.9%), belonging to the Firmicutes phylum, was also present to a lesser extent. The genus *Limnobacter* has been previously described as a heterotrophic thiosulfate-oxidizing bacterium (Lu et al. 2011, Spring et al. 2001), therefore, this genus might have been enriched due to the presence of sulfate (2 mM) in the low phosphate medium. Bacteria able to utilize thiosulfate like the genus *Pseudomonas* were shown to be versatile in their selection of electron donors and some of them can couple nitrate reduction to Fe(II) oxidation (He et al. 2023). Therefore, the genus *Limnobacter* may be capable of NRFeOx. Additionally, other abundant genera could be involved in N cycling such as the genus *Simplicispira*, which was previously described as a denitrifying bacterium (Peng et al. 2019, Siddiqi et al. 2020), while *Brevibacillus* is capable of both nitrification and denitrification (Joong et al. 2005). The relative abundance of the most abundant genera *Denitromonas* and *Limnobacter* fluctuated over time (between the different transfers; Fig. 4A). Although the microbial community analysis for each transfer was not done in triplicates, these fluctuations could still indicate a change in the bacterial community of the enrichment over time.

When the enrichment culture was incubated with acetate (0.5 mM), nitrate (1 mM), and Fe^2+^_aq_ (1 mM) at pH 6.5, nitrate and acetate consumption occurred simultaneously with Fe(II) oxidation for three consecutive transfers (Fig. 4B). During the first 5 incubation days, nitrate, Fe^2+^_aq_ and acetate consumption rates for the first transfer were 0.24 ± 0.00 mM NO_3_^−^/day, 0.09 ± 0.04 mM Fe^2+^_aq_/day and 0.14 ± 0.00 mM acetate/day, respectively. The initial Fe(II)/Fe_tot_ ratio was 0.73 ± 0.02, which decreased to 0.44 ± 0.04 within the first 7 days without further decrease after 7 days. Comparable rates were observed for the second and third transfer (Table S3). For all transfers, most of the Fe(II) oxidation happened within the first 7 days of incubation but the added Fe(II) was never fully oxidized. Based on the heterotrophic denitrification redox equation that couples nitrate reduction to N_2_ and acetate oxidation to CO_2_ (Albina et al. 2021), the amount of added acetate (0.68, 0.48, and 0.56 mM for transfers 1, 2, and 3, respectively) was sufficient to reduce 1.13, 0.79, and 0.93 mM of nitrate. This aligns well with the observed nitrate decrease (1.18, 0.76, and 1.06 mM NO_3_^−^ for transfers 1, 2, and 3, respectively). Moreover, the calculated acetate_oxidized_/ NO_3_^−^_reduced_ ratios (0.57, 0.63, and 0.53 for transfers 1, 2, and 3, respectively) were slightly lower than the ones that were obtained growing culture KS (acetate_oxidized_/ NO_3_^−^_reduced_ ratio of 0.7) under mixotrophic conditions with acetate (Tominski et al. 2018). We detected up to 0.32 ± 0.21 and 0.57 ± 0.09 mM of NO_2_^−^ (in transfers 1 and 3, respectively) meaning that ∼30% and ∼50% of the initially added NO_3_^−^ were converted to NO_2_^−^ (within the first 2 and 4 days of incubation for transfer 1 and 3, respectively). The bacterial community was capable of reducing NO_2_^−^ further since it did not accumulate over time. The presence of NO_2_^−^ suggests that Fe(II) oxidation could be partially due to chemodenitrification and not only by a direct enzymatic Fe(II) oxidation, similar as previously observed for the NRFeOx bacterium *Acidovorax* sp. BoFeN1 (Dopffel et al. 2021, Kappler et al. 2005, Muehe et al. 2009).

CLSM analysis showed that bacterial cells in the NRFeOx enrichment culture were associated with sphere-like structures consisting of EPS and mineral components as it can be seen in Fig. 4(C) and (D). Fe^2+^ was also found to be associated with minerals and EPS, the positive correlation between and Fe^2+^-minerals, Fe^2+^-EPS, and EPS-minerals is shown in Fig. S7. Under the light microscope, the inner sphere looked dark green (not shown), which could indicate mixed-valent Fe minerals as previously observed both in culture KS (Nordhoff et al. 2017) and *Acidovorax* sp. BoFeN1 (Pantke et al. 2012). These dark green spheres were surrounded by an orange halo (not shown) most were likely composed of Fe(III) minerals. This may be due to the initial transformation of vivianite in the medium into an amorphous mixed-valent Fe-phosphate, which then undergoes further oxidation (Miot et al. 2009). A more recent study showed that vivianite oxidation starts at the surface of the mineral particle (Metz et al. 2024), therefore explaining the orange halo observed around the mineral particles in our NRFeOx enrichment. Cell incrustation was not observed by CLSM as the DNA fluoroprobe did not show any correlation with the reflection channel, which indicates the mineral components (Fig. S7). Autotrophic NRFeOx cultures, such as culture KS, HP, and AG are known to avoid or at least minimize cell incrustation (Jakus et al. 2021a, Grimm et al. 2024, Straub et al. 2004), other NRFeOx cultures requiring acetate for growth, such as the mixotroph BoFeN1 or culture KS grown under mixotrophic conditions become encrusted by Fe(III) minerals. This suggests that Fe(II) oxidation occurs partly or entirely via abiotic processes involving NO_2_^−^ rather than enzymatically catalysed (Kappler et al. 2005, Nordhoff et al. 2017). The presence of Fe(II) minerals could mitigate cell incrustation and catalyse Fe(II) oxidation in *Acidovorax* sp. BoFeN1by acting as nucleation site for newly formed Fe(III) minerals (Cheng et al. 2022). The bacteria present in our culture might adapted to the high Fe^2+^_aq_ environment by producing EPS, as observed under CLSM, preventing Fe(III) minerals-cells incrustation (Chan et al. 2004) as also observed in culture KS (Huang et al. 2023a).

Overall the bacterial community of the NRFeOx estuarian enrichment was dominated by the *Denitromonas* genus and was shown to be capable of combining Fe(II) oxidation with NO_3_^−^ reduction. The enriched NRFeOx culture showed comparable acetate_oxidized_/ NO_3_^−^_reduced_ ratios, production of nitrite, and cell incrustation as observed in other NRFeOx cultures grown under mixotrophic conditions. To assess if the obtained NRFeOx enrichment can still be active under *in situ* conditions, the culture was grown at different pH and salinity values.

### Enrichment of NRFeOx from the reduced tidal sediment—pH and salinity effect

The NRFeOx enrichment culture, dominated by the genus *Denitromonas*, was incubated at pH 7.0, 6.7, and 6.2. These circumneutral pH values were chosen based on *in situ* geochemical data of the porewater collected from the reduced sediment layer (Bottaro et al. 2025). Triplicates were set up for each pH and the NRFeOx enrichment culture (at the 17th transfer) was incubated with 1 mM of NO_3_^−^, 2 mM of Fe(II), and 0.5 mM of acetate. Nitrate, acetate, and Fe^2+^_aq_ consumption as well as nitrite accumulation and Fe(II) oxidation extent were followed at each pH over time. At pH 6.2, nitrate and acetate were fully consumed within 3 days while at pH 6.7 and 7 full acetate and nitrate consumption was reached after 7 days (Fig. 4A). Acetate_oxidized_/ NO_3_^−^_reduced_ ratios (ca. 0.5 for each pH) were comparable to the ones obtained in earlier transfer stages (transfer 10). This delay in acetate and nitrate consumption as pH 7 compared to pH 6.2 was unexpected since denitrification and NRFeOx processes are favored at neutral pH (Foglar and Gašparac 2013, Straub et al. 1996). 16S rRNA gene copy numbers between the setups grown at different pH values were also comparable at the beginning of the experiment (Table S4) suggesting that the delayed nitrate and acetate consumption at pH 7 was not due to a difference in the number of inoculated bacteria. 16S rRNA gene copy numbers also increased comparably for each pH setup after within the 25 days of incubation. NO_2_^−^ was detected in the bottles incubated at pH 6.7 and 6.2 after 3 days (0.68 ± 0.06 and 0.79 ± 0.03 mM NO_2_^−^, respectively) and after 7 days at pH 7 (0.71 ± 0.13 mM NO_2_^−^). Overall, the extent of NO_2_^−^ production at pH 7 and 6.7 was similar and ∼70% of the initial NO_3_^−^ was converted to NO_2_^−^ while at pH 6.2 it was ∼80%. At pH 7 and 6.7, Fe^2+^_aq_ decrease was observed only after full nitrate/acetate consumption when NO_2_^−^ was also produced (at day 7). At pH 6.2, the Fe^2+^_aq_ decrease started at day 3 concomitantly with acetate and nitrate consumption. By quantifying the Fe(II)/Fe_tot_ ratios of the solid phase, we concluded that the decrease of Fe^2+^_aq_ observed in each setup was due to oxidation. Within the 25 incubation days, Fe(II)/Fe_tot_ ratios decreased form 0.93 ± 0.06 to 0.56 ± 0.05, from 0.84 ± 0.05 to 0.63 ± 0.05, and from 0.88 ± 0.02 to 0.71 ± 0.03 at pH 7.0, 6.7, and 6.2, respectively (Table S5). As ≥70% of the added NO_3_^−^ was converted to NO_2_^−^ within the first week of incubation, chemodenitrification was expected to be the main Fe(II) oxidation pathway at all tested pH values. By using literature stoichiometric equations coupling Fe^2+^_aq_ oxidation and NO_2_^−^ reduction to N_2_O or N_2_ (Sørensen and Thorling 1991, Tai and Dempsey 2009, Chen et al. 2020), the theoretical NO_2_^−^_reduced_/ Fe^2+^_aq oxidized_ ratios would be 0.50 (assuming FeOOH and N_2_O production at pH of 6.8) or 0.33 (assuming FeOOH and N_2_ production). Based on these equations we obtained NO_2_^−^_reduced_/ Fe^2+^_aq oxidized_ ratios of 0.84, 0.50, and 0.18 for the NRFeOx culture grown at pH 7, 6.7, and 6.2, respectively. The NO_2_^−^ values used for the calculation corresponded to the difference between the highest NO_2_^−^ concentrations detected and the amount of NO_2_^−^ still present at the end of the experiment. As Fe(II)/Fe_tot_ ratios of the solid phase decreased over time, we also based these calculations on the assumption that the Fe^2+^_aq_ decreased due to oxidation. The NO_2_^−^_reduced_/ Fe^2+^_aq oxidized_ ratio of 0.50 calculated at pH 6.7 matched exactly with the theoretical one assuming N_2_O formation indicating that NO_2_^−^ mainly abiotically reacted with the dissolved Fe^2+^_aq_. The higher ratio obtained at pH 7 suggested a different Fe^2+^_aq_/Fe(II) oxidation pathway. The theoretical NO_2_^−^_reduced_/ Fe^2+^_aq oxidized_ ratios calculation did account only for the dissolved Fe^2+^_aq_ phase and did not consider the reaction of NO_2_^−^ with solid-phase Fe(II) minerals. However, Fe(II) minerals, such as vivianite and siderite, are commonly present in NRFeOx cultures that grow in the LP medium and their precipitation extent increases at increasing pH (Postma 1980, Hegler et al. 2008, Nordhoff et al. 2017, Goedhart et al. 2022). Therefore, a higher Fe(II) mineral content was expected in the NRFeOx enrichment culture grown at pH 7 compared to pH 6.2 where most of the added FeCl_2_ (2 mM) remained in solution as Fe^2+^_aq_. The negative correlation between Fe^2+^_aq_ and pH based on our data can be seen in Fig. S8. Nitrite not only reacts with Fe^2+^_aq_ but can also abiotically oxidize Fe(II) minerals as vivianite (Lu et al. 2024, Miot et al. 2009) with chemodenitrification rates increasing at increasing pH (Chen et al. 2020) while contradictory results on the pH influence on chemodenitrification have been obtained by Dhakal et al. (2013). Despite these contradictory results, several studies consistently showed that the presence of Fe(II) minerals or mixed valent Fe minerals and the sorption of Fe^2+^_aq_ to newly formed Fe(III) minerals can increase chemodenitrification (Sørensen and Thorling 1991, Coby and Picardal 2005, Dhakal et al. 2013, Chen et al. 2020). Based on these previous observations, we conclude that chemodenitrification was favored under higher pH conditions and in the presence of higher Fe(II) mineral content resulting in increased Fe(III) precipitation and lower NO_2_^−^ accumulation (after 25 days of incubation) in the NRFeOx enrichment culture grown at pH 7. The lower NO_2_^−^_reduced_/Fe^2+^_aq oxidized_ ratio of the NRFeOx enrichment culture grown at pH 6.2 (compared to the theoretical values assuming N_2_ and N_2_O production) would complement this conclusion supporting the hypothesis that chemodenitrification decreased at lower pH and Fe(II) mineral content, explaining the lower Fe(III) mineral formation and higher NO_2_^−^ accumulation compared to pH 7 and 6.7.

**Figure 5.**
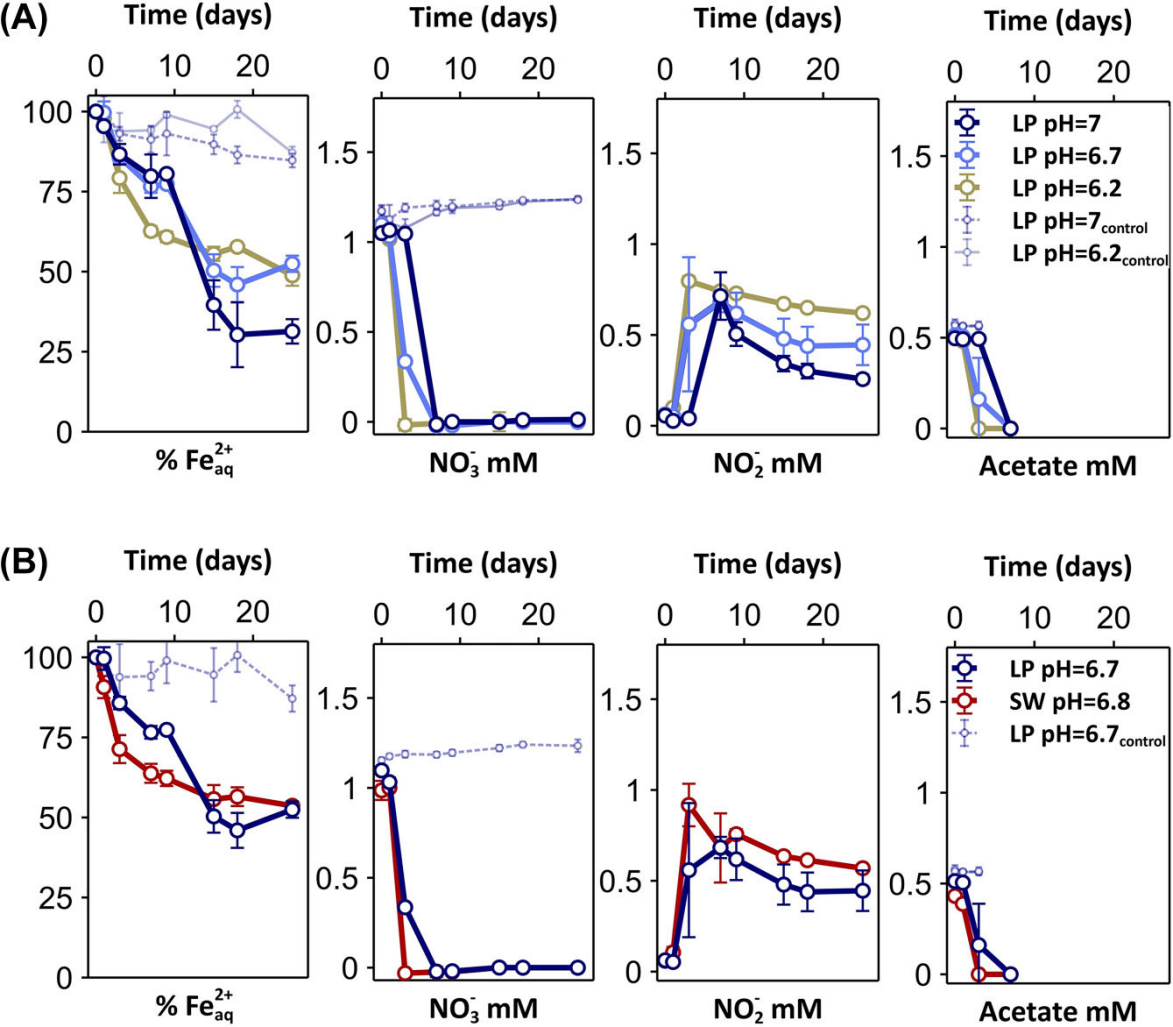
Geochemistry of the Río Tinto NRFeOx enrichment culture grown at different pH and salinity. Changes in NO_3_^−^, NO_2_^−^, Fe^2+^_aq_, and acetate in the enrichment culture over 25 days of incubation under different pH (A) and salinity (B). Geochemical data for the abiotic controls (no inoculum added) shown in lighter colors with dashed lines. (A) Shows data for the enrichment when it was inoculated (10%) in the low phosphate medium (indicated at LP) at three different pHs (7.0 in dark blue, 6.7 in light blue, and 6.2 in yellow). (B) shows data when the enrichment was inoculated (10%) in the seawater medium (SW) at pH 6.8 (red line) and geochemical were compared to the enrichment inoculated in the LP medium at a similar pH (6.7) to determine the effect of increased salinity on the NRFeOx enrichment. The Fe^2+^_aq_ data are expressed as percentages since the setups had different initial Fe^2+^_aq_ concentrations as discussed in section 2.6.

As shown in Fig. 5(A), NO₂^−^ was not fully consumed by the end of this experiment. By contrast, as discussed in section 3.5, NO₂^−^ did not accumulate in the NRFeOx enrichment culture grown for three generations at pH 6.5 in the LP medium. Based on the sequencing data from section 3.5, we hypothesize that the bacterial community may have shifted between the 10th transfer (used for the experiment in section 3.5) and the 17th transfer (used for the experiment discussed in the current section). Therefore, considering the consistent amount of added acetate, NO_3_^−^, Fe(II), inoculum, and pH ranges across all experiments, changes in the bacterial community over time may have influenced the culture´s ability to fully reduce NO_2_^−^.

Additionally, to simulate high tide conditions and test the salinity effect on the NRFeOx enrichment culture, the enrichment was grown in saltwater medium (medium composition is listed in Table S1) at pH 6.8. Nitrate, acetate, and Fe^2+^_aq_ consumption, as well as nitrite accumulation and Fe^2+^_aq_ oxidation extent, were followed over time and compared to geochemical data obtained by growing the same NRFeOx enrichment culture in LP medium at a comparable pH of 6.7 (Fig. 5B). In the NRFeOx enrichment culture grown in SW medium, nitrate and acetate were both consumed within the first 3 days of incubation and the calculated acetate_oxidized_/ NO_3_^−^_reduced_ ratio was also 0.5. Fe^2+^_aq_ decreased starting at day 3 concomitantly with acetate and nitrate consumption. NO_3_^−^ conversion to NO_2_^−^ in the enrichment grown in SW medium was up to 100% and on average the highest (0.92 ± 0.12 mM of NO_2_^−^ detected after 3 days of incubation) compared to the enrichment grown in the LP medium. The increased NO_2_^−^ production under salty conditions was also observed in the microcosms experiment (section 3.3). The decrease of Fe^2+^_aq_ under salt water conditions was linked to Fe(II) oxidation as the Fe(II)/Fe_tot_ ratio of the solid phase decreased from 0.88 ± 0.00 to 0.66 ± 0.03 within the 25 days of incubation, which matched what was observed in the NRFeOx enrichment culture grown in LP medium at pH 6.7 (from 0.84 ± 0.05 to 0.63 ± 0.05). These data provide additional evidence of pH being the main geochemical parameter controlling chemodenitrification processes in our NRFeOx enrichment culture. Moreover, the 100% conversion of NO_3_^−^ to NO_2_^−^ in the enrichment culture under salt water conditions indicated that chemodenitrification was the main Fe(II) oxidation pathway consistent with previous findings of (Huang et al. 2023b) and as demonstrated by the calculated NO_2_^−^_reduced_/ Fe^2+^_aq oxidized_ of 0.42.

Overall, we observed that the NRFeOx enrichment culture obtained from the reduced sediment layer in Río Tinto can be active and can adapt to fluctuating pH and salinity conditions. High salinity and lower pH conditions accelerated nitrate and acetate consumption as well as NO_2_^−^ production pointing towards chemodenitrification as main Fe(II) oxidation pathway under *in situ* conditions. Specifically, pH was shown as the major parameter controlling Fe(II) oxidation with Fe(III) mineral precipitation favored at neutral pH. The ∼100% conversion of NO_3_^−^ to NO₂^−^ under saltwater conditions in the presence of acetate, nitrate, and Fe(II) was systematically observed both in the enrichment cultures and microcosm experiment. Overall, these findings highlight the environmental importance of chemodenitification in controlling denitrification, Fe(II) oxidation, and, indirectly, HM immobilization in an environment that is characterized by strong geochemical fluctuations such as the Rio Tinto estuarian sediment.

## Funding

This work was funded by the German Research foundation. A.K. acknowledges infrastructural support by the DFG under Germany’s Excellence Strategy, cluster of Excellence EXC2124, project ID 390838134. We would like to thank the Institute for Medical Microbiology and Hygiene (MGM, where the NGS sequencing methods were performed) of the University of Tübingen with the support of the DFG-funded NGS Competence Centre Tübingen.

## Supplementary Material

fiaf083_Supplemental_File

## Data Availability

Research data will be uploaded to the FDAT Repository at the University of Tübingen. Raw sequencing data has been deposited at NCBI in the Sequence Read Archive (SRA) under BioProject accession number PRJNA1256346 (https://www.ncbi.nlm.nih.gov/bioproject/PRJNA1256346).
