# Supplementary material for: Impact of pH and salinity fluctuations on oxidation of Fe(II) by nitrate-reducing microorganisms enriched from the reduced tidal sediment of an extreme acidic river (Río Tinto, Spain)
Source: FEMS Microbiol Ecol. 2025 Aug 29;101(10):fiaf083. doi: 10.1093/femsec/fiaf083 (PMC12475565; doi:10.1093/femsec/fiaf083)
Supplement: fiaf083_Supplemental_File [file fiaf083_supplemental_file.pdf]

Impact of pH and salinity fluctuations on oxidation of Fe(II) by nitrate-reducing microorganisms enriched from the reduced tidal sediment of an extreme acidic river (Río Tinto, Spain)

MARTINA BOTTARO[1], SERGEY ABRAMOV[2], RICARDO AMILS[3], DANIEL STRAUB[4,5], SEBASTIAN KÜHNEL[1], MARIE MOLLENKOPF[1], SARA KLEINDIENST[2], MARTIN OBST[6]
ANDREAS KAPPLER[1,7*]

[1]Geomicrobiology, Department of Geosciences, University of Tübingen, Germany
[2]Institute for Sanitary Engineering, Water Quality and Solid Waste Management, University of Stuttgart, Germany
[3]Centro de Biología Molecular Severo Ochoa (CSIC-UAM), Universidad Autónoma de Madrid, Madrid, Spain
[4]Quantitative Biology Center (QBiC), University of Tübingen, Germany
[5]M3 Research Center, Medical Faculty, University of Tübingen, Germany
[6]Experimentelle Biogeochemie, BayCEER, University of Bayreuth, Germany
[7]Cluster of Excellence: EXC 2124: Controlling Microbes to Fight Infection, Tübingen, Germany

*Corresponding author:

Andreas Kappler (andreas.kappler@uni-tuebingen.de)

For submission to FEMS Microbiology Ecology

Supplementary Data

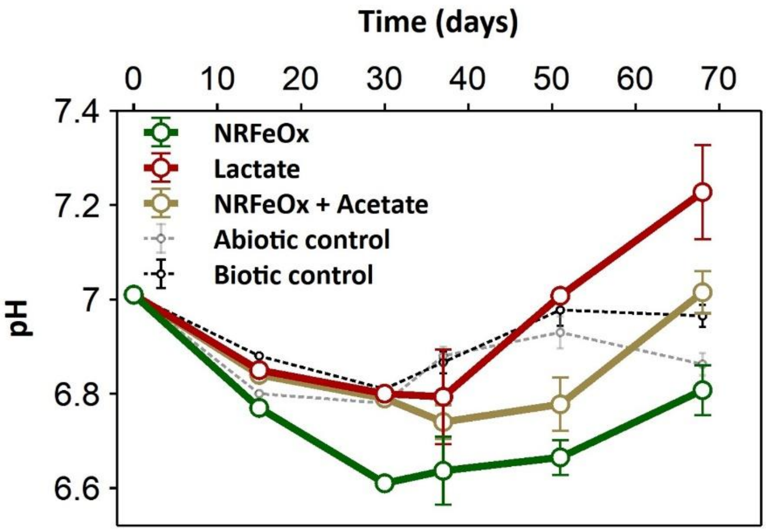

Fig. S1. pH variations over time in the microcosm experiments in the different setups and controls. Mean and standard deviation for three replicates.

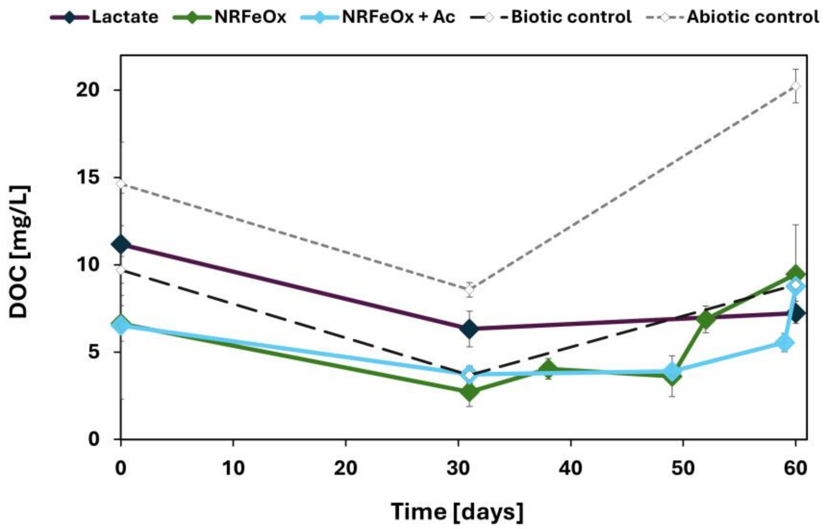

Fig. S2. pH variations over time in the microcosm experiments in the different setups and controls. Mean pH with standard deviation in error bars for three replicates.

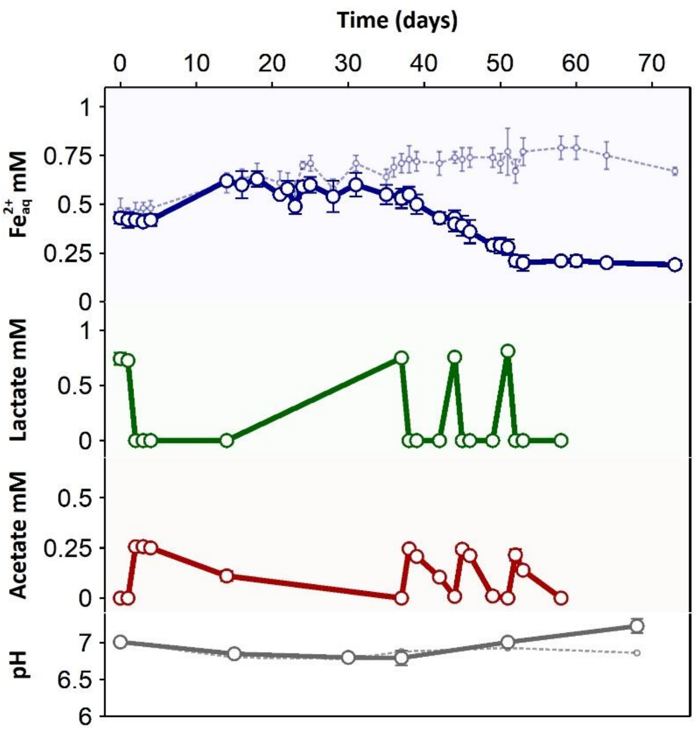

Fig. S3. Concentration of Fe^{2+}_{aq} (blue), lactate (red), acetate (yellow), and pH (grey) over time in the microcosm setup amended only with lactate. The abiotic control (no amendments) is shown in lighter colour with dashed lines. Mean with standard deviation in error bars for three replicates.

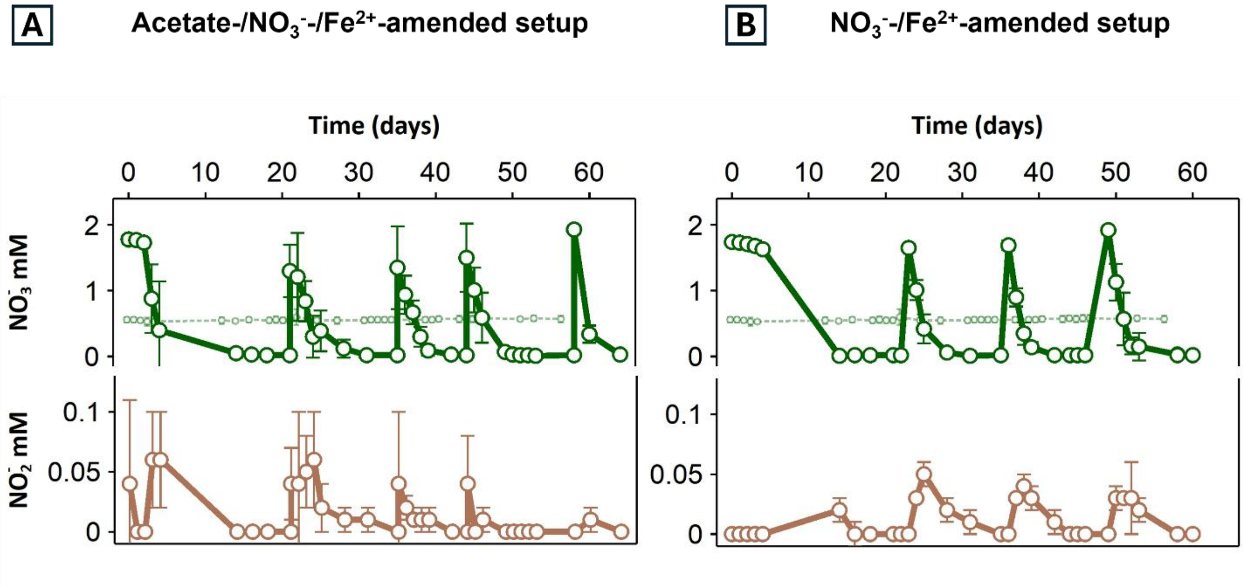

Fig. S4. Changes in NO_3^- (green) concentrations and production of NO_2^- (orange) in the acetate-/NO_3^--/Fe^{2+}-amended setup **(A)** and in the NO_3^--/Fe^{2+}-amended setup **(B)** over time. The abiotic control is shown as the smaller symbols and a dashed line. Mean with standard deviation in error bars for four replicates.

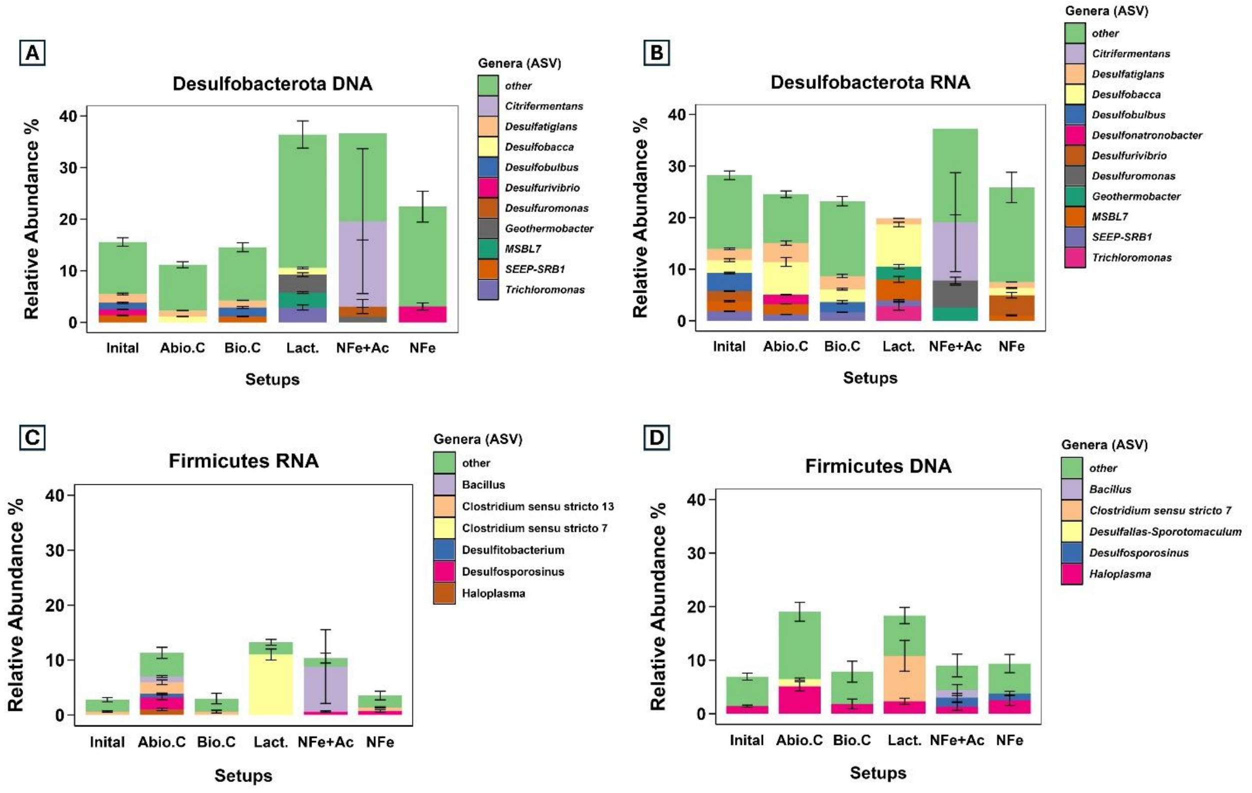

Fig. S5. DNA-based relative 16S rRNA gene relative abundances of the main genera belonging to the Desulfobacterota **(A, B)** and Firmicutes **(C, D)** enriched during the anoxic incubations in different setups. Genera below 1% (on average) were not included in the graph. Mean with standard deviation in error bars for three replicates.

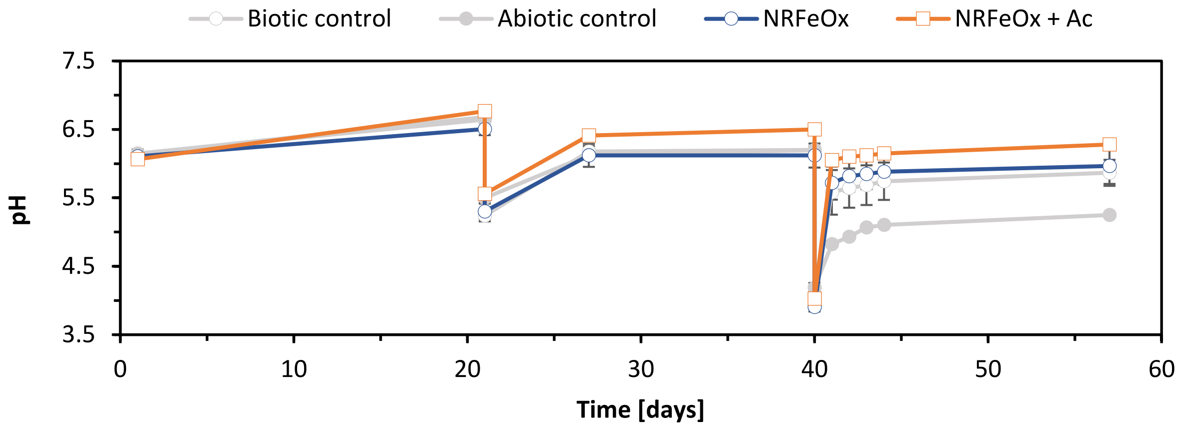

Fig. S6. pH changes in the acetate-/NO_3^--/Fe^{2+}- amended setup (orange), the NO_3^--/Fe^{2+}-amended setup (blue), and controls (grey) during the low tide simulation experiment (other geochemical data are shown in Fig. 3B).

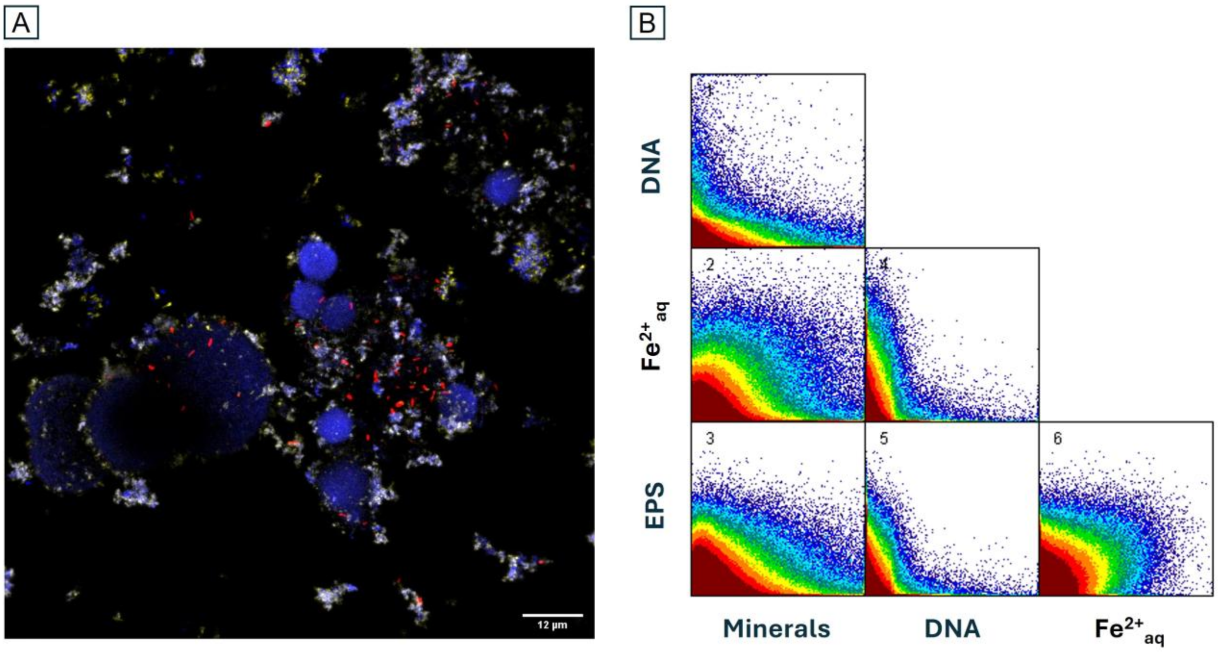

Fig. S7 (A) Maximum intensity projection of a CLSM image stack of the NRFeOx enrichment (of the same spot shown in Fig. 4) to visualize the EPS (in blue, SBA 647), DNA (in red, SYTO 40) and Fe^{2+}_{aq} (in yellow). The grey spots represent the areas where the culture was stained both with the EPS and Fe^{2+}_{aq} fluoroprobe. The staining procedure, image acquisition and treatment are described in section 2.7. **(B)** matrix of scatterplots obtained with the raw data of the different fluoroprobes/channels obtained with the ScatterJn plugin. Minerals (indicated as mineral components in the text) indicates the image acquired in the reflection channel. X and Y axis describe increasing signal intensities of the respective channels whereas the colour scale indicates the number of voxels of that combination.

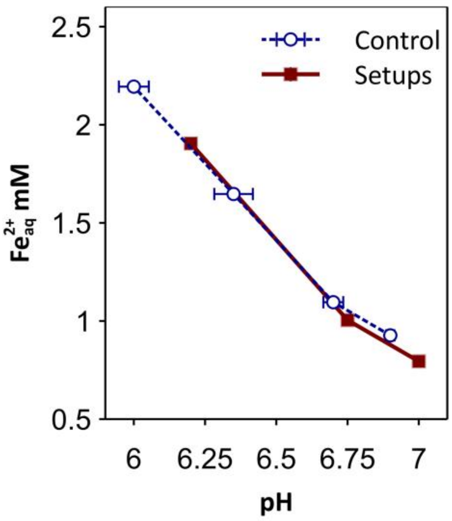

Fig. S8. Negative correlation between pH and Fe^{2+}_{aq} in the control (blue) and different setups at the beginning of the experiment (day 0). Mean with standard deviation in error bars for three replicates

Table S1 Elemental composition of the low phosphate medium (LP) and salt water medium (SW).

**LP Medium Salts**	**[g]**	**Buffer**	**[g]**	**[mM]**	**Additives**	**mL/L**
KH_2PO_4	0.14	$NaHCO_3$	1.85	22	Trace elements SL10	1
NaCl	0.2	N_2/CO_2 flushed			7 Vitamine solution	1
NH_4Cl	0.3				Selenite/Tungstate solution	1
$MgSO_4$ x 7 H_2O	0.5					
$CaCl_2$ x 2 H_2O	0.1					
MQ ad	1000					
**SW Medium Salts**	**[g]**	**Buffer**	**[g]**	**[mM]**	**Additives**	**mL/L**
					Trace elements SL10 7	1
					Vitamine solution	1
NaCl	17.3	NaHCO3	1.85	22	Selenite/Tungstate solution	1
$MgCl_2$ x 6 H_2O	8.6	N_2/CO_2 flushed,				
$MgSO_4$ x 7 H_2O	0.025					
$CaCl_2$ x 2 H_2O	0.99					
KCl	0.39					
KBr	0.059					
**KH_2PO_4**	**0.05**					
NH_4Cl	0.25					
MQ ad	1000					

Table S2 Element average concentrations of all the 250 ml bottles used for the microcosm experiment after mixing the low phosphate medium with the sediment slurry at the beginning of the experiment (see section 2.2). These data were obtained by ICP-MS measurement. The symbol ± indicates the standard deviation of each concentration.

Element	Average concentration (mg/L)
**Na**	1688.8±1021.0
**Mg**	118.7±10.0
**S**	105.5±17
**K**	75.929±6.2
**Ca**	51.131±7.5
**Cr**	0.068±0.008
**Mn**	0.127±0.0643
**Co**	0.002±0.001
**Ni**	0.007±0.0042
**Cu**	0.024±0.0032
**Zn**	9.881±0.587
**As**	0.498±0.080
**Se**	0.040±0.006
**Mo**	0.012±0.004
**Sb**	0.020±0.039
**Ba**	0.211±0.0159
**Pb**	0.183±0.011
**Th**	0.010±0.008
**U**	0.001±0.000

Table S3. Substrate consumption rates calculated for the NRFeOx enrichment culture grown for three consecutive generations. The symbol ± indicates the standard deviation of each concentration.

		Consumption rates		
**Generations**	**Days**	**Fe^{2+} mM/day**	**NO_3^- mM /day**	**Acetate mM /day**
1	5	0.09±0.04	0.24±0.00	0.14±0.00
2	5	0.11±0.00	0.15±0.00	0.10±0.00
3	6	0.03±0.00	0.18±0.00	0.09±0.00

Table S4. 16S rRNA (gene) copy numbers of the NRFeOx enrichment culture grown under different pH and salinity conditions at the beginning and end of the experiment. LP refers to low phosphate medium while SW refers to sea water medium. For each bottle, 2 mL of the bacteria culture were sampled for 16S rRNA (gene) copy numbers quantification with qPCR.

		**16S rRNA (gene) copy numbers**			
**medium**	**pH**	**Initial (day 0)**	**Standard deviation (day 0)**	**Final (day 25)**	**Standard deviation (day 25)**
**LP**	**7**	1.65E+03	7.67E+02	1.42E+05	2.06E+04
**LP**	**6.7**	1.51E+03	2.82E+02	1.30E+05	9.05E+03
**LP**	**6.2**	1.71E+03	3.97E+02	1.21E+05	2.87E+04
**SW**	**6.8**	8.52E+02	6.20E+02	5.83E+04	1.92E+04

Table S5. $Fe(II)/Fe_{tot}$ ratios in the mineral phase (pellet) of the NRFeOx enrichment culture grown under different pH and salinity conditions at the beginning and end of the experiment. LP refers to low phosphate medium while SW refers to sea water medium.

		$Fe(II)/Fe_{tot}$ ratios			
**medium**	**pH**	**Initial (day 0)**	**Standard deviation (day 0)**	**Final (day 25)**	**Standard deviation (day 25)**
**LP**	**7**	0.93	0.01	0.57	0.05
**LP**	**6.7**	0.85	0.05	0.63	0.05
**LP**	**6.2**	0.89	0.02	0.71	0.01
**SW**	**6.8**	0.89	0.01	0.66	0.03
**C_LP**	**7**	1.02	0.01	0.93	0.02
**C_LP**	**6.2**	1.03	0.00	0.96	0.11
**C_SW**	**6.8**	0.98	0.01	0.91	0.14
